# The Reciprocal Principle of Selectand-Selector-Systems in Supramolecular Chromatography †

**DOI:** 10.3390/molecules21111535

**Published:** 2016-11-15

**Authors:** Volker Schurig

**Affiliations:** Institute of Organic Chemistry, University of Tübingen, Auf der Morgenstelle 18, 72076 Tübingen, Germany; volker.schurig@uni-tuebingen.de; Tel.: +49-7071-62605

**Keywords:** supramolecular chromatography, electromigration methods, selectand, selector, reciprocal principle, combinatorial libraries, cyclopeptides, single-walled nanotubes (SWCNTs), fullerenes, cyclodextrins, calixarenes, congeners, enantiomers, molecular recognition, chiral recognition, chiral stationary phases

## Abstract

In selective chromatography and electromigration methods, supramolecular recognition of selectands and selectors is due to the fast and reversible formation of association complexes governed by thermodynamics. Whereas the selectand molecules to be separated are always present in the mobile phase, the selector employed for the separation of the selectands is either part of the stationary phase or is added to the mobile phase. By the *reciprocal* principle, the roles of selector and selectand can be reversed. In this contribution in honor of Professor Stig Allenmark, the evolution of the *reciprocal* principle in chromatography is reviewed and its advantages and limitations are outlined. Various *reciprocal* scenarios, including library approaches, are discussed in efforts to optimize selectivity in separation science.

## 1. Introduction

Separation approaches in highly selective supramolecular chromatography [1,2], with their most refined expression of stereochemical resolution [3], rely on the fast and reversible non-covalent association equilibrium between functionally and/or structurally complementary partners. The designations *selector* and *selectand* were introduced by Mikeš into chromatography to avoid ambiguities that may arise from the use of the notations *solvent-solute*, *ligand-substrate*, or *host-guest* [4]. The new terms were coined in analogy to Ashby’s cybernetic *operator-operand* terminology [5]. This terminology extends to all supramolecular interactions such as antibody-antigen, receptor-substrate, metal ion-ligand etc. In chromatographic partitioning systems, selectors are employed to separate mixtures of selectands which are added to the mobile phase. The selector is either present as a stationary phase or as an additive to the liquid mobile phase. In order to find an optimized selector for a pair of selectands, the role of selector/selectand can be reversed by the *reciprocal* principle. One molecule of the selectand is now used as the stationary phase and an array of potential structural related selectors are now employed as analytes in the mobile phase. The best identified candidate is then used as the optimized lead stationary phase for the target selectands to be separated. Thus the *reciprocal* recognition principle applies to selective separation system when selectors and selectands change their role and belong to two or more forms of congeners, analogues, isomers etc. In the special case of chiral recognition, both enantiomers of selectand and selector must be available. This excludes proteins [6], carbohydrates [7] and antibodies [8], although they represent versatile chiral stationary phases (CSPs). Pertinent examples will be described in the following Sections.

## 2. Origin of the *Reciprocal* Principle in Chromatography

**Figure 1:**
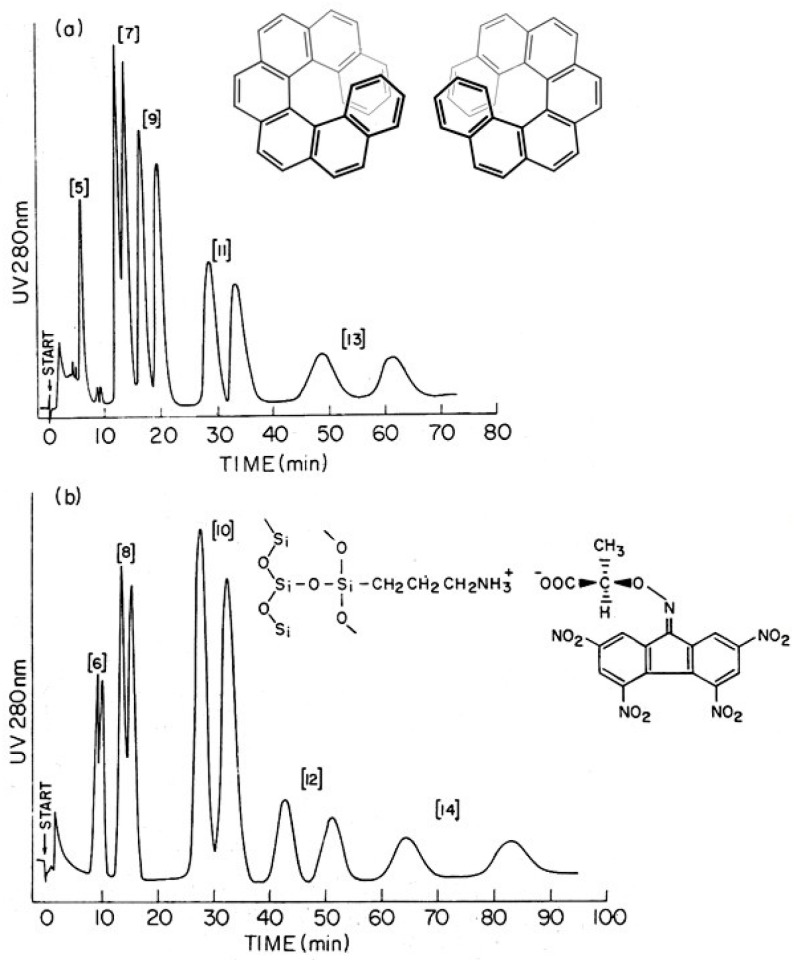
Enantiomeric separation of racemic [5]–[14]helicenes on (*R*)-(−)-2-(2,4,5,7-tetranitro-9-fluorenylideneamino-oxy)propionic acid (TAPA) linked to silica gel [4]. (**a**) odd numbers; (**b**) even numbers. Insertions: [7]helicene and TAPA.

**Figure 2:**
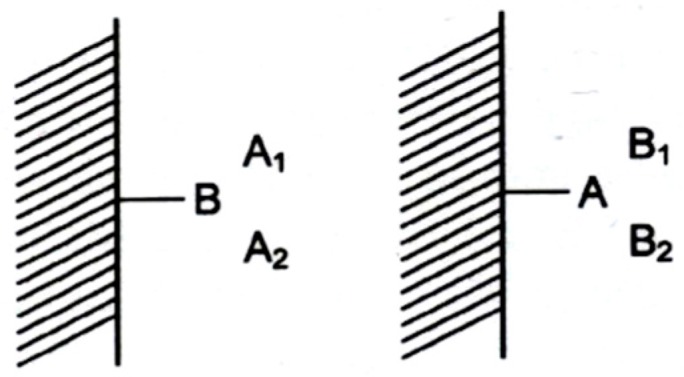
The *reciprocal* supramolecular recognition principle. If the selector B separates the selectands A_1_ and A_2_, than the selector A is expected to separate the selectands B_1_ and B_2_. A_i_ and B_i_ are homologous compounds, congeners or isomers (including enantiomers).

Following the enantioseparation of [5]–[14]helicenes on the optically active charge-transfer agent *R*-2-(2,4,5,7-tetranitro-9-fluorenylideneamino-oxy)propionic acid (TAPA, Figure 1) or its 2-butyric acid homologue (TABA) [9,10,11], Mikeš speculated that the function of the selector and the selectand could be reversed, i.e., optically active helicenes could be used as resolving agents for racemic compounds [4]. Thus, in a *reciprocal* fashion, if the selectands A1 and A2 are chromatographically separated on the selector B1 (or B2), the selectands B1 and B2 are separated on the selector A1 (or A2) as well, whereby the respective pairs A1/A2 and B1/B2 refer to isomers (e.g., stereoisomers) or belong to members of homologous series of compounds (Figure 2). Indeed, it was subsequently demonstrated that *P*-(+)-hexahelicene-7,7′-dicarboxylic acid disodium salt could be used as the chiral stationary phase (CSP) as π-donor selector for the enantioseparation of *N*-2,4-dinitrophenyl(DNP)-α-amino acid esters as π-acceptor selectands by HPLC [12].

The principle of *reciprocity* by inverting the role of selectand and selector had first been demonstrated via the differentiation of enantiomers by chiral solvating agents (CSA) in NMR spectroscopy [13]. Pirkle et al. stated: *the enantiomers of secondary and tertiary alcohols may be caused to have nonequivalent nmr spectra through the use of appropriate optically active amines as solvents. The converse of this phenomenon also obtains; amine enantiomers may have nonequivalent nmr spectra in optically active alcohols* [14] and Pirkle and Hoover concluded that the roles of an enantiomeric solute and a CSA *may be interchangeable* for a given pair of compounds [15]. The *reciprocal* principle was later extended by Pirkle et al. to enantioselective liquid chromatography [16,17]. The researchers pointed out that one can take one chiral stationary phase (CSP) to develop others: *if a CSP derived from (+)-A retains (+)-B, then a CSP comprised of immobilized (+)-B should, using the same interactions, selectively retain (+)-A* [18,19]. Or put in other words by Pirkle and Pochapsky: *This* ‘*bootstrapping*’ *method of designing reciprocal CSPs from compounds that themselves resolve on existing CSPs is based on the premise that if two molecules show mutual chiral recognition, then it does not matter which of the two is bound to a stationary support for that recognition to occur. This is in practice not strictly correct, for the nature of attachment to the CSP often affect chiral recognition. Within limits, however, reciprocity is a useful guide to CSP design* [20]. The latter important restrictions was repeatedly stressed by Pirkle et al.: *The two situations are not* ‘*mirror images*’ *and the relationship is only approximate. The manner of immobilization, for example, has some bearing upon the efficacy of the chiral recognition process* [19]. Thus deviations from the *reciprocal* principle may arise from the environment of the selector in the stationary phase, e.g., as the result of immobilization and the presence of a spacer linking the selector to a chromatographic matrix [21].

**Figure 3:**
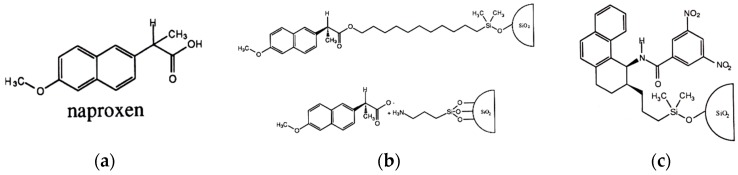
(**a**) Structure of naproxen; (**b**) A single enantiomer of naproxen attached covalently or ionically on silica; (**c**) The optimized CSP Whelk-O1 (commercialized by Regis Chemical Co., Morton Grove, IL, USA). Reprinted (adapted) with permission from [23].

A vivid example of the de novo design of a CSP for a particular target chiral selectand by the *reciprocal* principle involved a series of CSPs developed for the separation of the enantiomers of the non-steroidal anti-inflammatory drug (NSAID) naproxen [22,23]. By the so-called *immobilized guest method*, a single enantiomer of naproxen (Figure 3a) was immobilized to form a naproxen-derived CSP (Figure 3b) which was then used to identify a potential candidate from an array of test racemates as a de novo enantioselective naproxen selector [23]. This *reciprocal* study also identified the structural requirements for enantioselective recognition for the target racemate naproxen. The tailor-made Whelk-O1 CSP (Figure 3c) not only showed the highest enantioseparation factor for naproxen (α = 2.25) but also enantioseparated structurally related NSAIDs as well as a host of different racemates by a combination of face-to-edge, and face-to-face π-π interaction as well as hydrogen bonding [22,23]. The Whelk-O1 CSP offered a broad spectrum for enantioseparations of aromatic selectands. The enantioseparation of *bis*-, *tris*- and *hexakis*-adducts of C_60_ has also been achieved by HPLC on the Whelk-O1-CSP (Section 4).

Some preliminary work on the use of the *reciprocal* principle has been reported for the design of pyrethroid specific CSPs [24]. In a preliminary attempt to design novel CSPs for the resolution of the stereoisomers of the insecticide cypermethrin, the principle of *reciprocal* interaction has been considered via the following rationale: ‘*If a CSP based on a pure enantiomer (+)-A resolves a racemate (+/−)-B*, *then a single pure enantiomer (+)- or (−)-B should resolve (+/−)-A*. *This is true if no major point of interaction is changed when attaching B to silica*’. It should be mentioned that by employing the CSP (−)-A instead of (+)-A, a reversal of the elution order of (−/+)-B would obtain. Likewise by employing the CSP (−)-B instead of (+)-B, again a reversal of the elution order of (−/+)-A would obtain.

The biologically most active (1*R cis S*) stereoisomer of cypermethrin has been structurally modified by an allylic group, followed by free radical addition or hydrosilylation reaction on two different silanes and linking (i) a short-chain monofunctional unit and (ii) a long-chain trifunctional unit, respectively, to silica. As cypermethrin can be resolved on *N*-(3,5-dinitrobenzoyl)-phenylglycine CSP, the *reciprocal* interaction of the cypermethrin CSP with racemic *N*-3,5-dinitrobenzoyl-phenylglycinepropylamide was claimed [24]. The actual objective to use the cypermethrin-derived CSPs to identify and optimize the interaction with racemic test compounds from which a single enantiomer will then be bound to silica to afford a cypermethrin-specific CSP still awaits its realization [24].

### 2.1. Capillary Electromigration Methods (CE)

In enantioselective capillary electromigration methods, the non-racemic chiral selector is added to the background electrolyte as part of the mobile phase. The method of enantioseparation of charged selectands with neutral selectors is referred to capillary zone electrophoresis (CZE), whereas the method of enantioseparation of neutral selectands with charged selectors, entailed with self-mobility, is referred to electrokinetic chromatography (EKC) [25]. Chankvetadze argued that from a mechanistic point of view there is no principle difference whether the selectand or the selector is charged in electromigration methods [26]. The philosophy behind this concept has been stated as follows: *if a neutral chiral selector can resolve the enantiomers of a charged chiral analyte, then the enantioseparation of the neutral chiral analyte should also be possible with the charged chiral selector* [26]. Thus another type of the *reciprocal* principle applies to electromigration methods. Whereas the enantioseparation of neutral chiral selectands on charged chiral selectors were at first considered not possible in earlier studies of EKC, it was later successfully realized based on the *reciprocal* concept. Thus positively charged cyclodextrin derivatives [25,27] and negatively charged cyclodextrin derivatives [27,28,29,30] were used for the enantioseparation of a multitude of *neutral* chiral compounds [31,32].

Another *reciprocal* principle in the achiral electromigration domain has not yet been realized, but may be anticipated. Native α-, β-, γ-cyclodextrin [33] and, more recently, δ-cyclodextrin [34] have been used as an added mobile phase selector for molecular association with various positively or negatively charged selectands in CZE. By inverting their role, a charged selector may be able to separate various cyclodextrin congeners preceding δ-CD (α-, β-, γ-) and, more importantly, congeners following up δ-CD (>δ).

**Figure 4:**
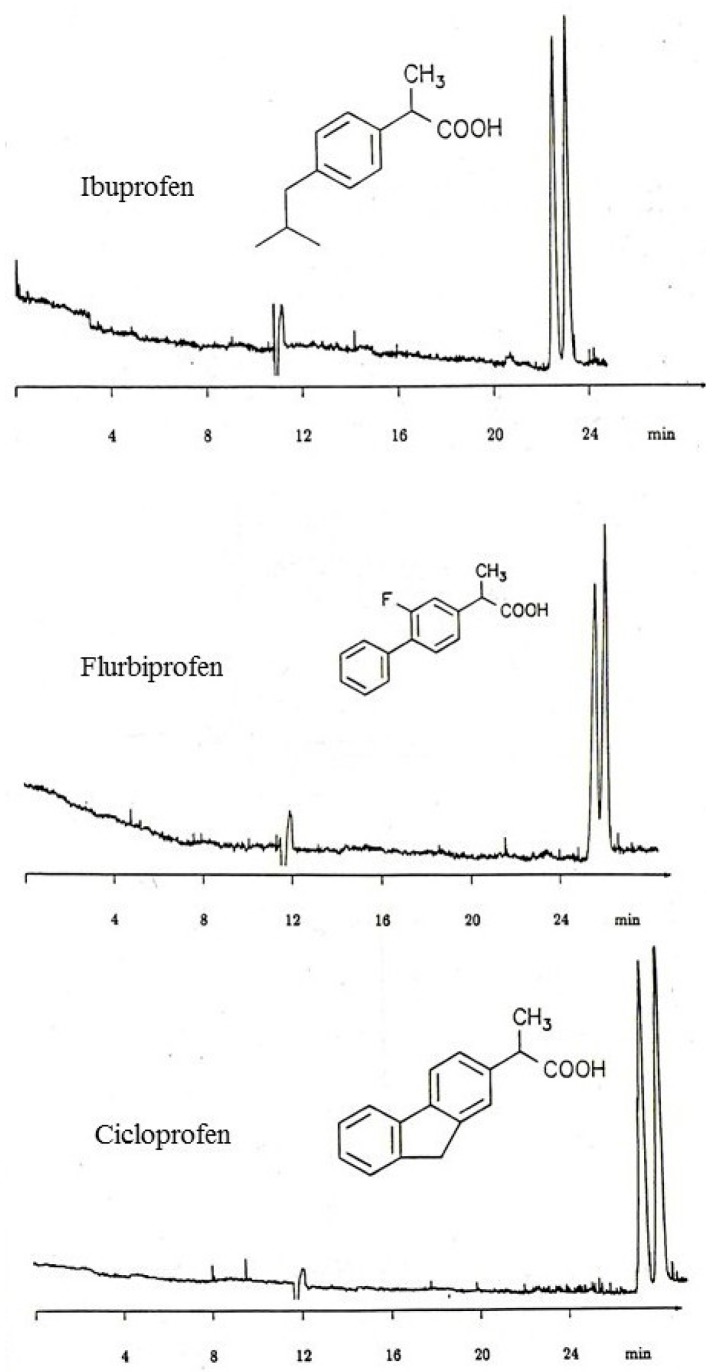
Enantioseparation of profens by electrochromatography on Chirasil-β-Dex coated and immobilized (d*_f_* = 0.20 μm) on an 80 cm (effective) × 50 μm I.D. fused-silica capillary at 20 °C and 30 kV. Buffer: 20 mM Tris/HCl at pH 7. Reproduced from S. Mayer, Doctoral Thesis, University of Tübingen, Tübingen, Germany, 1993.

In enantioselective capillary electrochromatography (*enantio*-CEC), a non-racemic chiral selector is used as CSP coated on the inner capillary wall. As the first examples, racemic 1-phenylethanol [35,36] and some non-steroidal anti-inflammatory drugs (NSAIDs, Figure 4) were enantioseparated on Chirasil-Dex (permethylated β-cyclodextrin chemically linked to polydimethylsiloxane and thermally immobilized on the inner wall of a 80 cm (effective) × 50 μm I.D. capillary column).

**Figure 5:**
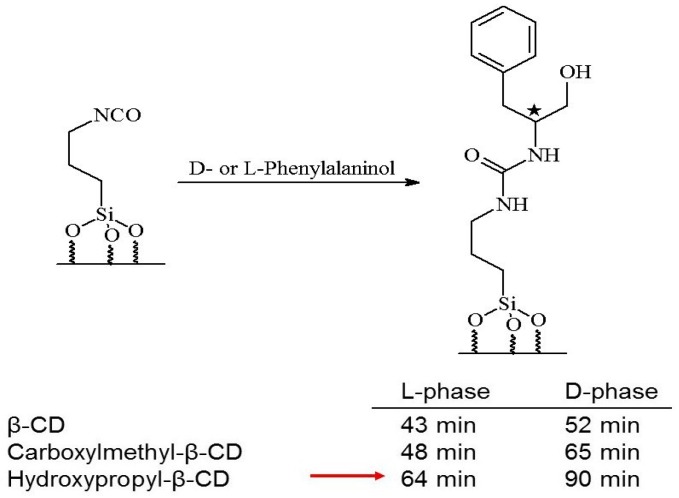
Retention time differences of three modified β-cyclodextrins (β-CD as mono-2.6-dimethylphenylcarbamate) on silica-bound d- and l-phenylalaninol by the CEC-experiment (50 mM ammonium acetate, pH 6.5, 15 kV, column: 68 cm eff. × 50 µm I.D.) [37].

In order to find the best cyclodextrin selector for a given racemic analyte by the *reciprocal* approach, the target molecule may now be used as CSP, whereas the modified cyclodextrin is added to the mobile phase. However, as the cyclodextrins are only available in one enantiomeric form (comprised of d-glucose building blocks) *two* columns coated with the l- and d-target molecule, respectively, are required. As shown in Figure 5 the highest enantioselectivity for phenylalanilol was observed with hydroxypropyl-β-cyclodextrin due to a large retention time difference on the two mirror image CSPs [37].

Although not related to the *reciprocal* principle, the following approach is of interest in its own right. A dual chiral recognition system can be obtained when one cyclodextrin selector is used as the CSP whereas another one is added to the mobile phase (combination of CEC and EKC). As the enantioselectivities of the bonded and free CDs were opposite, the incremental addition of the sulfonated β-cyclodextrin to a Chirasil-Dex-coated capillary led to an inversion of the elution order of racemic 1,1’-bi-2,2’-naphthol-hydrogenphosphate (20 mM borate/phosphate (pH 7) buffer, at 30 kV) [29].

### 2.2. Combinatorial Library Approaches in Achiral Supramolecular Chromatographic Systems

The *reciprocal* principle has been used for the rational design of an optimized selector for molecular recognition. Another optimization principle is based on the use of combinatorial selector libraries aimed at identifying the prime selector for a given enantioselective separation system. This approach can also be combined with the *reciprocal* principle [38,39]. It has generally been accepted that the screening of libraries of selectors anchored to solid supports allows the identification of key structural elements responsible for host/guest interaction and molecular recognition through binding assays with soluble selectands. Combinatorial methods have been classified into two major categories. One is based on the synthesis of mixtures and another is based on the parallel synthesis of pure library components (also called the high-throughput approach). In the former approach, a mixture of library components is synthesized and screened simultaneously for the desired property. In the parallel library approach, each library component is synthesized and screened individually. The advantage of the parallel approach is that the library is generally better characterized, whereas the random library technique is advantageous in that a large number of components can be synthesized and screened more efficiently. A classification of the various combinatorial approaches for the preparation and use of CSPs for enantioseparations has been summarized [40].

Peptide libraries have been used to optimize selectors that bind specifically to a desired target protein in the area of protein purification. Solid phase ‘one-bead-one-peptide’ (OBOP) parallel combinatorial libraries have been successfully employed to search for affinity ligands for large proteins. Thus the immobilized linear hexapeptide Try-Asn-Phe-Glu-Val-Leu was identified as a potential host for the purification of S-protein by affinity chromatography [41]. Also an affinity resin containing the peptide ligand Phe-Leu-Leu-Val-Pro-Leu has been developed for the purification of fibrinogen. The selector was identified by screening the solid-phase combinatorial peptide library using an immunostaining technique [42]. In a reversed *reciprocal* fashion, affinity chromatography employing the immobilized S-protein was used for the screening of affinity peptide selectands from a soluble peptide library consisting of octamers with glycine (Gly) at both termini of each peptide, i.e., Gly-X-X-X-X-X-X-Gly. The six variable centre positions were constructed using random sequences of the six l-amino acids Tyr, Asn, Phe, Glu, Val and Leu. Peptides that were retained specifically on the immobilized S-protein column were eluted by 2% acetic acid. The peptides in the acid eluate were further separated using reversed-phase HPLC. Each separated peptide fraction was collected and the peptide sequences deconvoluted by mass spectrometry (MS/MS). The screenings of the peptide library resulted in twelve affinity peptides and eight out of them contained the essential sequence Asn-Phe-Glu-Val [43].

### 2.3. Combinatorial Cyclopeptide Library Approaches in Chiral Capillary Electrophoresis

**Figure 6:**
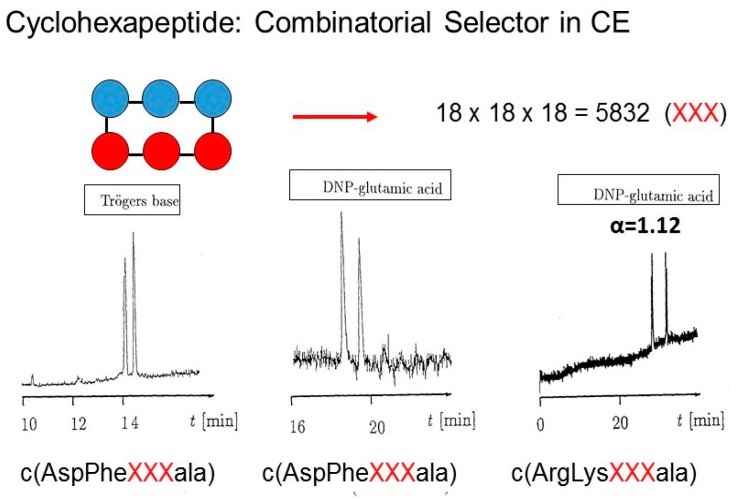
Enantioseparation of racemic Tröger’s base and DNP-d,l-glutamic acid with combinatorial cyclohexapeptides by CE. Conditions: 10 mmol cyclopeptide library *cyclo*(OOXXXO) in phosphate buffer pH 7.4 (20 mmol). Capillary: 50 cm (effective) × 50 μm I.D., 20 kV (left, middle) and 10 kV (right), detection at 260 nm (left) and 340 nm (middle and right) [44].

Combinatorial cyclohexapeptide libraries were used as novel multicomponent chiral additives for enantioseparation in capillary electrophoresis (CE) by Jung and Schurig et al. in 1996 [44]. It was inferred that the time-consuming screening of a multitude of individual potential chiral selectors can be avoided by employing a whole selector library. The random library approach is then followed by deconvolution steps. The sub-libraries with reduced heterogeneity can then be employed to identify the most enantioselective selectors. In general, cyclopeptide libraries of high molecular diversity can be obtained by including structural variations. By using the nearly 200 commercially available amino acids of defined chirality, 200^6^ = 64 × 10^12^ individual cyclohexapeptides are in principle accessible. The number of cyclopeptides can further be increased by varying the ring size and by including non-peptidic components, derivatives with side chain and backbone modifications, and selected building blocks with inverted chirality [44]. Three libraries consisting of 18^3^ = 5832 individual cyclohexapeptides were synthesized as a random mixture and were characterized by electrospray mass spectrometry [44]. Each library had the composition *cyclo*(O-O-X-X-X-O) and consisted of three defined positions (O) and three randomized positions (X) which represented a mixture of 18 of the common l-α-amino acids except cysteine and tryptophan. To aid ring closure, one position (O) consisted of unnatural d-alanine (ala). When added to the mobile phase in CE, the libraries *cyclo*(Asp-Phe-X-X-X-ala), *cyclo*(Arg-Lys-X-X-X-ala) and *cyclo*(Arg-Met-X-X-X-ala), respectively, enantioseparated Tröger’s base and *N*-2,4-dinitrophenyl (DNP)- or Fmoc-d,l-glutamic acid (α = 1.04–1.13) (Figure 6) [44].

The result raised the question whether all 18^3^ individual components (which are present in the library at high dilution and probably in a non-equimolar ratio) possess the same or, more likely, different enantioselectivities with individual ‘losers and winners’. It may even be conceived that some members exert an opposite elution order of the selectand thus reducing the overall enantioselectivity. Cooperative effects between the individual selectors were believed to be absent [44]. To identify the prime selector in the library, Chiari et al. used an interesting deconvolution strategy [45].

This approach was performed in two steps: (i) the synthesis of sublibraries with a progressively increased number of defined positions and (ii) the evaluation of their ability to act as chiral selectors in CE for a set of DNP-d,l-amino acids. First of all, Chiari et al. deconvoluted the library *cyclo*(Arg-Lys-X-X-X-β-Ala) by substituting d-Ala (ala) of the library *cyclo*(Arg-Lys-X-X-X-ala) of Jung and Schurig et al. [44] by achiral β-alanine (Table 1, S.1.1) [45]. Then a number the sublibraries S.2.1, S.2.2 and S.2.3 were prepared, whereby proline was deliberately selected for one of the positions X as it facilitates hexapeptide cyclization. However, only proline in position four (Table 1, S.2.2) exerted a high enantioselectivity of the sublibrary.

**Figure 7:**
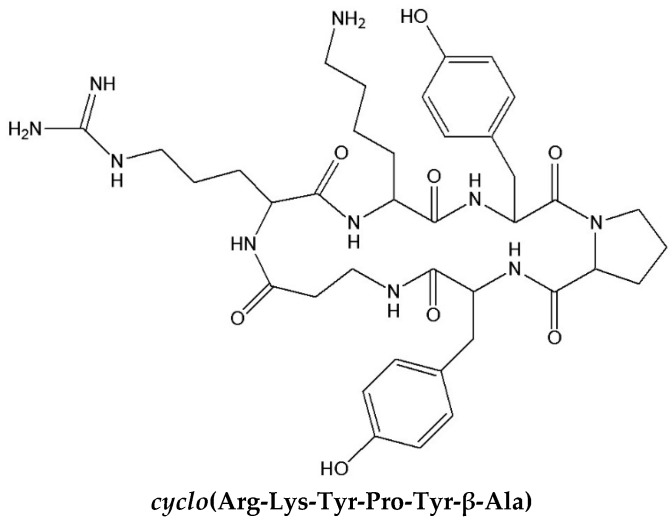
The optimized cyclohexapeptide library component, deconvoluted by Chiari et al. [45].

**Figure 8:**
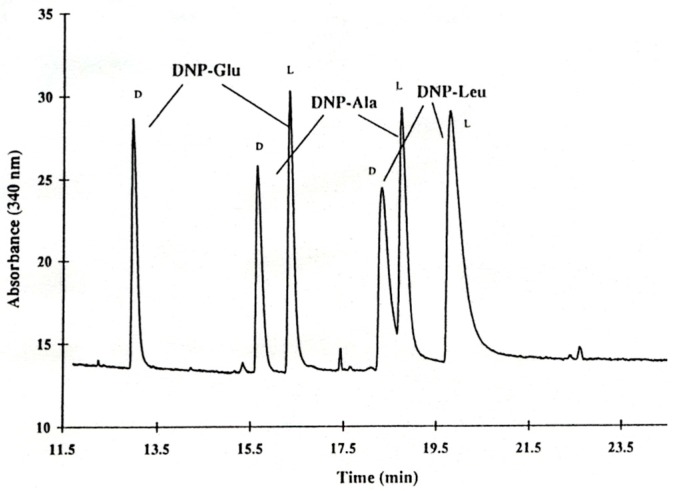
Enantioseparation of d,l-DNP-amino acids in a running electrolyte containing *cyclo*(Arg-Lys-Tyr-Pro-Tyr-β-Ala) (10 mM in 20 mM sodium phosphate buffer, pH 7.0) as chiral mobile phase additive (CMPA) in CE. Column: 57 cm (effective) × 50 μm I.D.; −20 kV, 15 °C. Reprinted with permission from [45]. Copyright (1998) American Chemical Society.

The optimized library *cyclo*(Arg-Lys-X-Pro-X-β-Ala) (S.2.2) was then semipreparatively resolved into three fractions by reversed-phase HPLC. The second fraction exerted the highest enantioselectivity and amino acid analysis revealed that this fraction contained the hydrophobic amino acids Val, Met, Ile, Leu and Phe. This gave rise to the synthesis of the six sublibraries S.3.1–S.3.6. (Table 1). The sublibrary *cyclo*(Arg-Lys-Tyr-Pro-X-β-Ala) (S.3.5) was selected for the further deconvolution, since Tyr contains an aromatic system useful for detection purposes. Now the six sublibraries S.4.1–S.4.6 were synthesized (Table 1). In a similar fashion position 5 was optimized by a preceding HPLC separation into three fractions and again identifying hydrophobic amino acids as being essential for enantioselectivity. Phe and Tyr in position 5 provided the highest enantioselectivity and hence the final defined cyclohexapeptide selector (Figure 7) has been selected for the enantioseparation of DNP-d,l-glutamic acid (α = 1.24) (Figure 8) [45].

The overall deconvolution process required the synthesis and the evaluation of 15 sublibraries instead of the 54 syntheses considered by a classical procedure of serial deconvolution [46].

In a follow up study, several cyclo*hexa*peptide and cylco*hepta*peptide analogues of the identified library members were studied as chiral selectors in countercurrent capillary electrophoresis [47]. At the operating pH of 7.0, all of the selectors bear a positive charge, while the analyte bears a negative charge. A poly(acrylamide-co-allyl amide of d-gluconic acid-co-allylglycidyl ether) was coated onto the capillary to suppress the EOF to ensure that the analyte and selector would migrate in opposite directions by counter-current CE. The following changes were made in the deconvoluted cyclohexapeptide *cyclo*(Arg-Lys-Tyr-Pro-Tyr-β-Ala). (i) Tyr in position five was substituted by Tyr(3-NO_2_), Phe, Phe(4-NO_2_) and Trp; (ii) Tyr in position three was substituted by positively charged Lys, negatively charged Glu and neutral Leu; (iii) Ala was inserted into *cyclo*(Arg-Lys-Tyr-Pro-Tyr-β-Ala) in the subsequent positions 1–6 to yield conformationally flexible cyclo*hepta*peptides with positional variety. All positively charged cyclo*hexa*peptides are effective chiral selectors for the enantioseparation of DNP-α-amino acids with resolutions factors *R_s_* = 1–5. The charge in position 3 did not have any influence on enantioselectivity of DNP-AAs. However, all of the cyclo*hepta*peptides failed to resolve DNP-amino acids [47]. This finding suggested that the size and rigidity of the cyclopeptide system was important for ensuring chiral discrimination. In subsequent NMR studies it was recognized that the presence of aromatic moieties carrying electron-withdrawing substituents, i.e., nitro groups, were essential for enantiorecognition via π-π stacking interactions and amide/aromatic *n*-π interactions [47,48].

**Figure 9:**
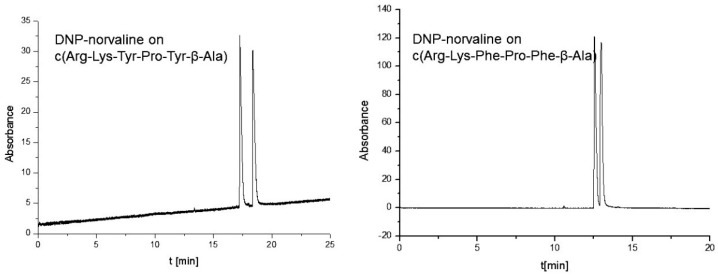
Influence of the change of Tyr for Phe in the cyclohexapeptide for enantioseparation of norvaline (conditions as in Figure 8) [49].

Based on the optimized *cyclo*(Arg-Lys-Tyr-Pro-Tyr-β-Ala) cyclohexapeptide selector deconvoluted by Chiari et al. [45], Jung and Schurig et al. also screened related cyclopeptides with small structural variations [49]. Thus aromatic tyrosine was replaced by phenylalanine and tryptophane in position 3 and 5. In both cases the enantioselectivity was reduced for several DNP-amino acids (Table 2, Figure 9) and the choice of the aromatic amino acid had a pronounced effect on enantioselectivity. When the five-membered ring of proline was opened, the rigid structure of the cyclohexapeptide was released and accompanied by ring extension. Thus when proline was substituted by 6-aminohexanoic acid in *cyclo*(Arg-Lys-Tyr-*Pro*-Tyr-β-Ala), no enantioseparation at all was observed for various DNP-amino acids [49]. Enantioselectivity also broke down when the amino acid sequence in *cyclo*(Arg-Lys-Tyr-*Pro*-Tyr-β-Ala) was changed to *cyclo*(Arg-*Pro*-Lys-Tyr-Tyr-β-Ala) and *cyclo*(Arg-*Pro*-Tyr-Lys-Tyr-β-Ala). Thus the sequence ‘*aromatic amino acid-proline-aromatic amino acid*’ (Tyr-*Pro*-Tyr) was essential for chiral recognition. Finally the ring size of the cyclopeptide was probed (Table 3) [49]. The highest enantioselectivity was observed with cyclo*hexa*peptides. In agreement with results of De Lorenzi et al. [47], enantioselectivity was lost for conformationally flexible cyclo*hepta*peptides. Surprisingly, this drop was even more pronounced with cyclo*penta*peptides (Table 3). When the cyclohexapeptide *cyclo*(Arg-Lys-Tyr-Pro-Tyr-β-Ala) was opened up to the linear peptide Arg-Lys-Tyr-Pro-Tyr-Lys by exchanging β-Ala for Lys no enantioselectivity for DNP-amino acids was observed [49]. Thus the cyclo*hexa*peptide *cyclo*(Arg-Lys-Tyr-Pro-Tyr-β-Ala) identified by Chiari et al. (Figure 7) [45] indeed represents the most favourable selector which could not be optimized further.

### 2.4. Reciprocal Principle in Combinatorial Approaches Utilizing Cyclopeptides—A Failure and a Success

**Figure 10:**
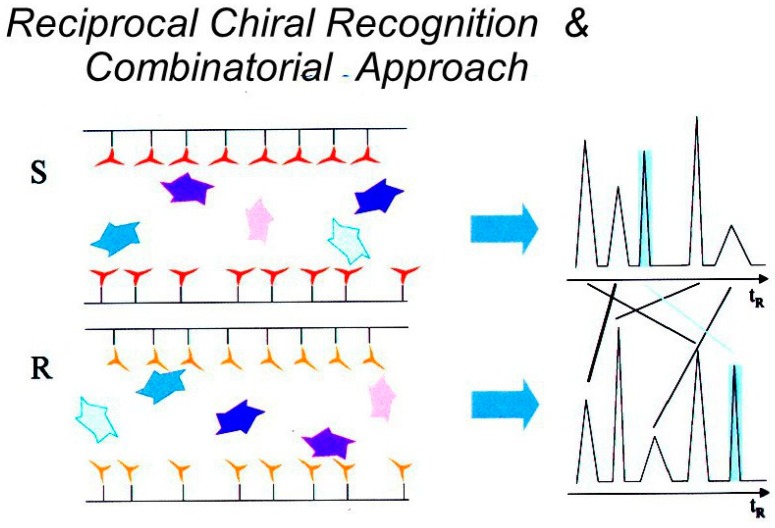
Illustration of the identification of an optimal selectand in a small library exhibiting the highest enantioselectivity toward a chiral selector used either in the *S*- or *R*-configuration as revealed by the largest difference of its retention (earmarked by the pale blue colour at the right).

Instead of a deconvolution strategy, a *reciprocal* LC approach has been considered by Jung, Schurig et al. to identify an enantioselective hit component in the deconvoluted library (Figure 10) [49].

Since the cyclopeptide library exists only in one enantiomeric form (obtained from l-amino acids) two different columns containing the target racemate separately in the l- and d-form, respectively, are required. The sub-library component present in the mobile phase which exhibits the largest difference in its retention on the two mirror-image selectors, should also show the highest enantioselectivity in the *reciprocal* system when used as chiral selector for the racemic non-bonded candidate used as selectand (Figure 10).

**Figure 11:**
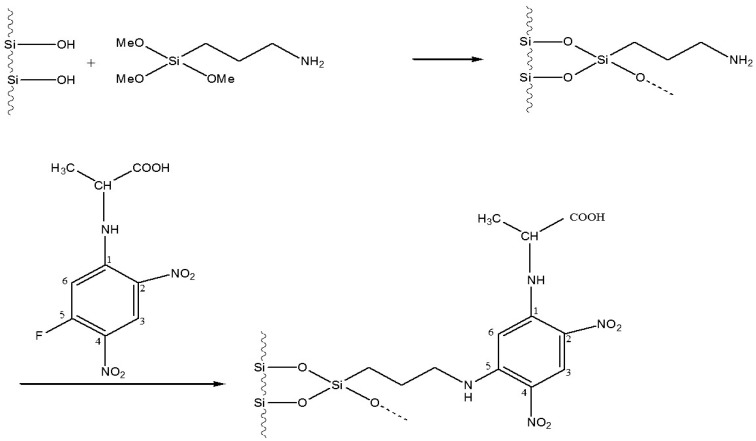
Linking of l- or d-DNP-alanine to silica (5 µm) via the amine route [49].

**Figure 12:**
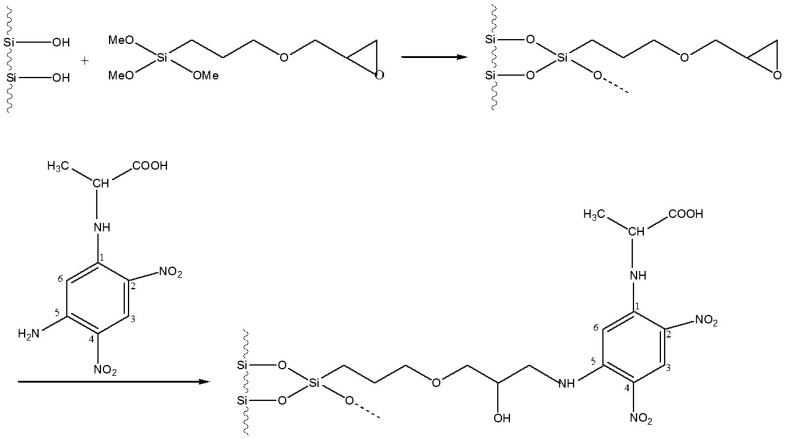
Linking of l- or d-DNP-alanine to silica (5 µm) via the epoxide route [49].

In initial experiments l- or d-DNP-alanine silica (Figure 11 and Figure 12) was filled into 100 µm I.D. capillaries. *Cyclo*(Arg-Lys-Tyr-Pro-Tyr-β-Ala) was selected as the analyte as it shows high enantioselectivity toward racemic DNP-alanine (Figure 8) [45]. Micro-HPLC measurements were performed in the reversed phase mode (H_2_O), in the organic polar mode (methanol/acetonitrile) and in the normal phase mode (toluene, *n*-hexane/2-propanol). However, no measurable retention time differences for the two mirror image stationary phases were observed. Also the coating of 50 µm I.D. capillaries with l- or d-5-fluoro-2,4-DNP-alanine in the presence of aminopropyltrimethoxysilane in toluene and the investigation of *cyclo*(Arg-Lys-Tyr-Pro-Tyr-β-Ala) by pressure-supported open-tubular capillary electrochromatography (OT-CEC) did not reveal differences in retention times as expected from the *reciprocal* principle [49]. This unexpected result reinforces the limitation of the *reciprocal* principle as enantioselectivity may be reduced by non-specific interactions on silica. This might be circumvented by end-capping of silanol groups or elongation of the linker categories [38,39].

Since the cyclohexapeptide *cyclo*(Arg-Lys-Tyr-Pro-Tyr-β-Ala), used as chiral mobile phase selector in CE, enantioseparates DNP-glutamic acid (Figure 8), the *reciprocal* principle was also tested by adding l-DNP-Glu in a first run, and d-DNP-Glu in a second run, to the background electrolyte in the CE experiment. The corresponding elution time of the added cyclohexapeptide was determined by indirect UV-detection. Unexpectedly also no striking difference of the elution time of the cyclohexapeptide was observed for the mirror-image situation of the two experiments between 15–20 °C and at voltages of 10–25 kV, again reinforcing the limitation of the *reciprocal* concept [49].

For screening a mixture of a combinatorial library for its best selector, also a *reciprocal* approach was developed by Li et al. in line with Figure 10 [50]. The model study concerned the optimized enantioseparation of (1-naphthyl)leucine ester with a small peptide library (4 × 4 = 16) containing Leu, Ala, Gly and Pro and four different *N*-acyl components. In a *reciprocal* fashion, l-(1-naphthyl)leucine ester was first immobilized and used as CSP for the enantiomeric peptide libraries obtained from l- and d-amino acids, respectively. l- and d-sublibraries, exhibiting the largest retention differences on the l-configurated CSP, were deconvoluted and the best selector for the racemate was identified [50]. The optimized peptide Leu-Gly was then attached to silica. It enantioseparated racemic (1-naphthyl)leucine ester with an enantioseparation factor α = 3. Thus it was indeed demonstrated that CSPs can be developed by a *reciprocal* chromatographic approach using enantiomeric libraries.

### 2.5. On-Bead Library Combinatorial Approaches

**Figure 13:**
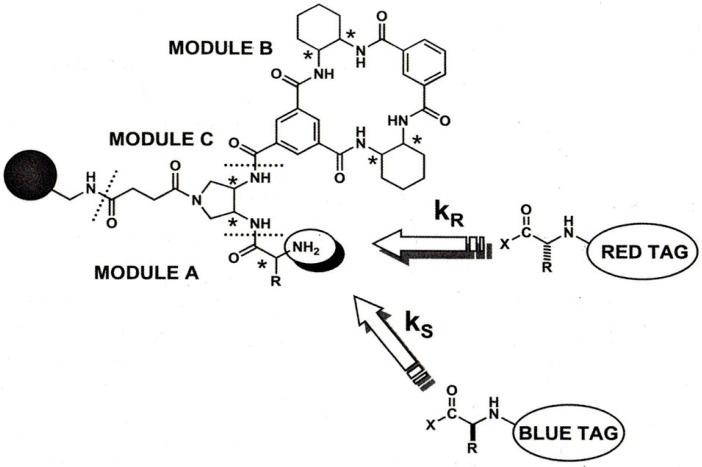
Structure of the multimodal receptor array and kinetic resolution of differently dye-tagged solid-supported amino acids from a 60-member library. Module A contains 15 different d- and l-amino acids, module B contains *RR* or *SS* diamine enantiomers, and the receptor module C contains *RRRR* or *SSSS* enantiomers resulting in 15 × 2 × 2 = 60 different library members [51]. Figure 13 reproduced with permission from [52].

For the enantioselective screening of a target racemate, Weingarten et al. used a resin library containing 60 members of a multimodal receptor which were bonded to polystyrene beads (100 μm) (Figure 13) [51]. The chemically encoded, solid-phase supported library was generated by a mix-and-split protocol employing three classes of chiral building blocks (Figure 13). The rationale behind this non-chromatographic screening method was stated as follows: *If a tightly binding and highly enantioselective* (e.g., *R_s_* > 10) *resin or other solid material were readily available, then it would be possible to resolve compounds by simply stirring the racemate with such a resin and filtering.* The screening method employed differently coloured enantiomeric target molecules that were labelled with differently coloured dyes. Thus a quasi-racemate included the blue l-α-amino acid derivative and the red d-α-amino acid derivative. Proline was selected as the test compound. The idea was to treat an equimolar mixture of these coloured probe molecules with a library of chiral selectors on synthetic beads in which each bead carried a different chiral selector (one-bead-one-selector, OBOS). It was reasoned that a highly enantioselective binding would yield beads that were either red or blue, whereas non-enantioselective binding would yield beads that were brown. Thus the best selector was directly visualized through a two-colour differential binding screening. The reddest and bluest beads found with each probe were then picked and decoded to determine the structures of their associated chiral selectors. Finally the leading resolving resins were individually resynthesized on a gram scale and again treated with excess red and blue proline enantiomers. Subsequent treatment with NaOMe and HPLC quantification of the released dyes provided a determination of the enantiomeric excess (*ee*) of the proline derivatives bound to the beads. The identified enantioselective resolving resin was then tested via its ability to kinetically resolve racemic proline ester in solution [51].

### 2.6. On-Bead Combinatorial Approach of Individual Library Members

**Figure 14:**
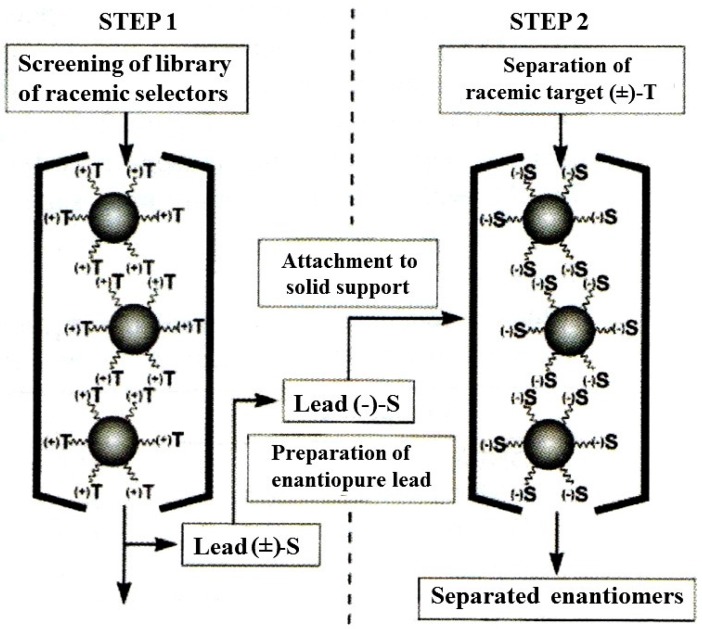
*Reciprocal* combinatorial approach to the optimization of a CSP. Reproduced with permission of the Royal Society of Chemistry from [55].

Fréchet et al. used an on-bead combinatorial approach to the rational design of optimized CSPs for HPLC. At first a library of 3 × 12 = 36 l-α-amino acid anilides (consisting of 3 α-amino acids and 12 aromatic amines) was synthesized in solution, then attached to functionalized macroporous polymeric methacrylate/dimethacrylate beads which were packed into a ‘library column’ and tested for the enantioseparation of the target analyte. The lead selector of the library, i.e., l-proline-1-indananilide, employed for the HPLC enantioseparation of the target racemate *N*-(3,5-dinitrobenzoyl)-leucine diallylamide, was identified by a deconvolution process using 11 ‘sublibrary columns’ of lower diversity, each of which containing a CSP with a reduced number of library components [53,54]. Fréchet et al. also combined the library approach and the *reciprocal* principle for the development of enantioselective CSPs for HPLC [55,56]. Thus according to Figure 14, a single enantiomer (+)-T of the target racemate was at first immobilized onto a polymeric support and the resulting CSP was used for the HPLC screening of sublibraries of racemic compounds that have been prepared by parallel combinatorial synthesis. The best enantioseparated member of this library (±)-S is then prepared as the single enantiomer (−)-S, coupled to a solid support and used in the second step for the required enantioseparation of the target racemate (±)-T. This *reciprocal* methodology has been exemplified for the target racemate *N*-(3,5-dinitrobenzoyl)-leucine and the parallel library components of racemic 4-aryl-1,4-dihydropyrimidines obtained in a one-pot three-component reaction (Biginelli dihydropyrimidine three-component condensation reaction) [56]. Hydrogen-bonding and π-π interactions played a dominant role in chiral recognition.

Libraries of potential chiral selectors have also been prepared by the Ugi three-component reaction of β-lactams and screened for their enantioselectivity by using the *reciprocal* approach involving a CSP comprised of the immobilized model target compound *N*-(3,5-dinitrobenzoyl)-l-leucine [57]. The lead candidate identified from the library of racemic phenylamides of 2-oxo-azetidine acetic acid derivatives was subsequently synthesized in bulk. The up-scaled racemate was then resolved by chiral preparative HPLC and one enantiomer was immobilized on a solid support. The obtained CSP showed enantioselectivities of α = 3 for various amino acid derivatives. Interestingly, the enantioselectivities observed were even higher than those obtained during the *reciprocal* screening on the immobilized target compound *N*-(3,5-dinitrobenzoyl)-l-leucine [57].

### 2.7. Batch-Screening of Peptide Libraries and the Reciprocal Principle

**Figure 15:**
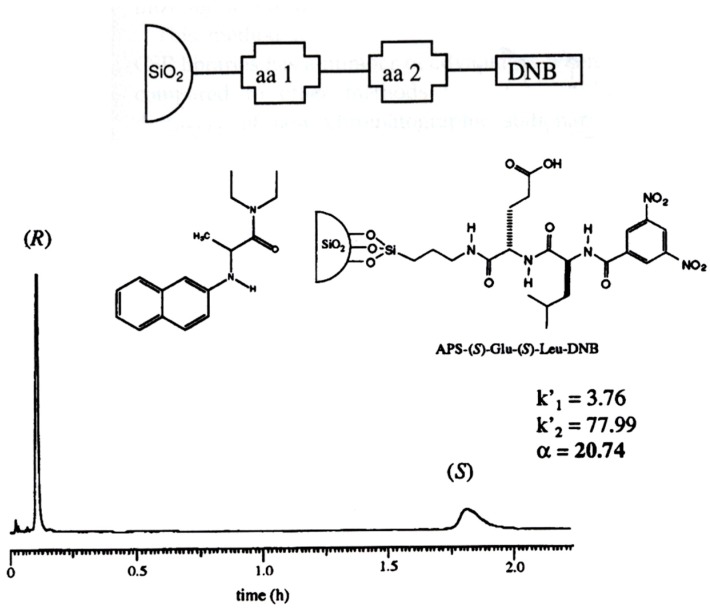
**Top**: DNB-dipeptide libraries used as CSP for the enantioseparation of *N*-(2-naphthyl)alanine diethylamide. **Bottom**: The optimized aminopropylsilica-bonded l-Glu-l-Leu-DNB selector enantioseparates the test racemate with high enantioselectivity. Column: 4.6 mm × 250 mm I.D., eluent: 2-propanol/*n*-hexane (20/80 *v/v*). Reprinted with permission from [61]. Copyright (1999) American Chemical Society.

Previous studies by Pirkle, Hyun et al. had shown that the enantiomers of racemic *N*-(3,5-dinitrobenzoyl)-peptide selectands are enantioseparated on a number of CSPs [58]. It was therefore reasoned that in a *reciprocal* fashion *N*-(3,5-dinitrobenzoyl)-peptide CSPs should be capable of enantioseparating various racemic compounds [59]. In an off-column batch-screening approach, Welch et al. probed the selector-selectand interaction directly, rather than resorting to a bonded analogue of the selectand used as CSP and packed into a column [60,61]. Thus the individual components of a parallel library of 50 silica-supported *N*-(3,5-dinitrobenzoyl)-dipeptide CSPs were prepared by solid phase synthesis on aminopropylsilica (APS) particles [59]. Whereas the ‘aa1’ position (Figure 15, top) consisted of the five d-α-amino acids Phg, Val, Pro, Gln and Phe, the ‘aa2’-position (Figure 15, top) was comprised of the same five α-amino acids in the l- and d-form, respectively, giving rise to 50 different dipeptides. The members of the library were subsequently screened for the enantioselective recognition of the test racemate *N*-(2-naphthyl)alanine diethylamide by an efficient batch-screening process. Thus individual vials containing approximately 50 mg of the candidate CSPs were placed into an autosampler and a dilute solution of the target racemate was added to each vial. The concentration (1 mL; 10^−5^ M in 2-propanol/*n*-hexane (20/80, *v/v*)) of the analyte solution was low enough to avoid saturation of the available interaction sites on the CSP. During equilibration using a rotary platform shaker, the individual enantiomers were partitioned between the solid and liquid phases of the vial. After equilibration, enantioselective HPLC of 50 μL injections of the supernatant solution in each vial afforded two peaks for the individual enantiomers. A 1:1 ratio revealed no enantioselectivity of the adsorbent, whereas ratios up to 1:2.72 indicated the presence of an enantioselective adsorbent. It was found that the homochiral combinations (DD) of the library components exhibited a higher enantioselectivity than the heterochiral combinations (LD) [60]. The presence of steric bulk in the ‘aa 2’ position and of hydrogen-bonding groups in the ‘aa1’ position proved to be important for enantiomeric discrimination of the test racemate. APS-bonded d-Gln-d-Val-DNB, d-Pro-d-Val-DNP and d-Gln-d-Phe-DNP were identified as the leading enantioselective selectors for racemic *N*-(2-naphthyl)alanine diethylamide. Based on these findings, an improved library of individual dipeptides was designed. It included amino acids with side chain functional groups such as Glu, His, and Arg, Whereas the ‘aa1’ position (Figure 15, top) consisted of the seven l-α-amino acids Gln, Asn, Ser, His, Asp, Arg and Glu, the ‘aa2’-position (Figure 15, top) was comprised of the l-α-amino acids Val, Leu, Ileu, Phe, Tyr, *tert*-Leu and Trp and the d-α-amino acids Val, Leu, Ileu, Phe, Tyr and Trp, respectively, giving rise to 91 different dipeptides. *N*-(2-naphthyl)alanine diethylamide was enantioseparated on APS-bonded l-Glu-l-Leu-DNP with the high enantioselectivity of α = 20.74 (Figure 15, bottom) [61]. Up to 100 mg of the compound could be enantioseparated using an analytical column (4.6 mm × 250 mm I.D.) in a single injection. The versatile approach of Welch et al. permitted the identification of the necessary structural requirements for chiral recognition by comparing the relative performance of various CSPs in the library. The enantioselective library approach, commercialized jointly by Regis Technology and MediChem Research [reported in: *Chem. Eng. News* **1999**, *11*, p. 101], may also prove useful for the discovery of improved selectors for other types of supramolecular chromatographic interactions.

An improved strategy for the evaluation of common CSPs by the straightforward single-vial-equilibration-approach of Welch et al. was based on rapid LC/MS screening of isotopically labelled *pseudoenantiomers* present in the supernatant instead of the HPLC analysis of the enantiomers [62].

Concurrently, another batch-wise screening method was described by Wang and Li [63]. The approach was almost identical to that of Welch et al. with the exception that the incubation was performed with the selector attached to a polymeric resin, instead of a chromatographic support. The reliability of the established stepwise solid-phase Merrifield-type peptide synthesis was considered as an advantage. However, it was also recognized that the screening result would not necessarily correlate with the chromatographic experiment as no chromatographic support was used for the batch screening step [38,39]. Crucial elements of the approach were the *reciprocity* of chromatographic separation and the use of enantiomeric libraries. Thus, a potential chiral selector from the parallel library was linked onto a solid-phase resin. The racemic analyte in the proper solvent was then allowed to equilibrate with this potential selector on the resin. The enantiomeric ratio of the analyte in the supernatant was analysed after the equilibration period by circular dichroism. A selective adsorption of one of the two enantiomers to the resin was indicative of an efficient chiral selector. It was then linked onto a chromatographic support, and its enantioselectivity toward the target analyte was evaluated. The feasibility of the parallel library screening procedure was compared with the model system studied earlier of the chiral HPLC enantioseparation of *N*-(1-naphthyl)leucine ester using a 16-member small library [50].

Bluhm et al. simplified the *reciprocal* system by using only one library (all-l) which was equilibrated on two different columns containing the immobilized target racemate (1-naphthyl)leucine ester either in the l- or enantiomeric d-form, followed by incubation of each CSP with a mixture library [64]. The adsorbed members of the library on the two columns were washed off and analysed by a reversed phase HPLC assay. Components with the same retention time but different intensities in both chromatograms represented an optimized selector. Its chemical structure was then determined by LC-MS or LC-MS-MS. In the *reciprocal* fashion, the optimized candidate, i.e., immobilized *N*-3,5-dinitrobenzoyl-l-leucine ester, was used as CSP to enantioseparate racemic *N*-(1-naphthyl)leucine ester with the high enantioseparation factor α = 12 [64]. In an optimized study, a 200-member parallel dipeptide library was created and screened with 10-undecenyl-*N*-(1-naphthyl)leucinate [65]. The library was prepared using Fmoc-protected α-amino acids with amino-methylated polystyrene (AMPS) resin as solid support. The library contained three modules. Modules 2 and 3 consisted of ten different l-α-amino acids, whereas the *N*-terminating module 1 was an acetyl group or a 3,5-dinitrobenzoyl (DNB) group. Each single library member was incubated with a solution of racemic 10-undecenyl-*N*-(1-naphthyl)leucinate. After equilibration, the supernatant was collected and assessed for enantiomeric excess *ee* by circular dichroism (CD).

In another study, an 81-member dipeptide library was evaluated for the target compound containing a stereogenic phosphorus atom, i.e., *tert*-butyl-1-(2-methylnaphthyl)phosphane oxide [66]. The member with the highest enantioselectivity was DNB-d-Asn-l-Thr-Abu-AMPS (Abu = 4-amino-butanoic acid). It was attached to aminopropylsilica and used as CSP for the target racemate in HPLC. However, the enantioselcetivities between the batch-experiment and the chromatographic experiment did not match due to the different solid supports. Therefore a longer linker had to be employed to immobilize the chiral selector onto silica to observe an enantioseparation factor of α = 3.2 for the racemic phosphine oxide. A 121-member peptide library was also tested for the enantioseparation of atropisomeric 1,1′-bi-2,2′-naphthol. A trityl-protected di-asparagine selector (Fmoc-Asn(Trt)-Asn(Trt)) yielded a separation factor of α = 7.2 [67]. By the *reciprocal* principle, it can be assumed that the target racemate 1,1′-bi-2,2′-naphthol, for which a highly enantioselective peptide has been identified, could also be used as a CSP for the enantioseparation of the racemic peptides.

Another high throughput screening protocol was proposed for chiral selector discovery. It was modeled after the protocol for biological screening of candidate drugs from chemical libraries. The procedure was based on target distribution between an aqueous phase and an organic phase [68].

## 3. Single-Walled Carbon Nanotubes, SWCNTs

**Figure 16:**
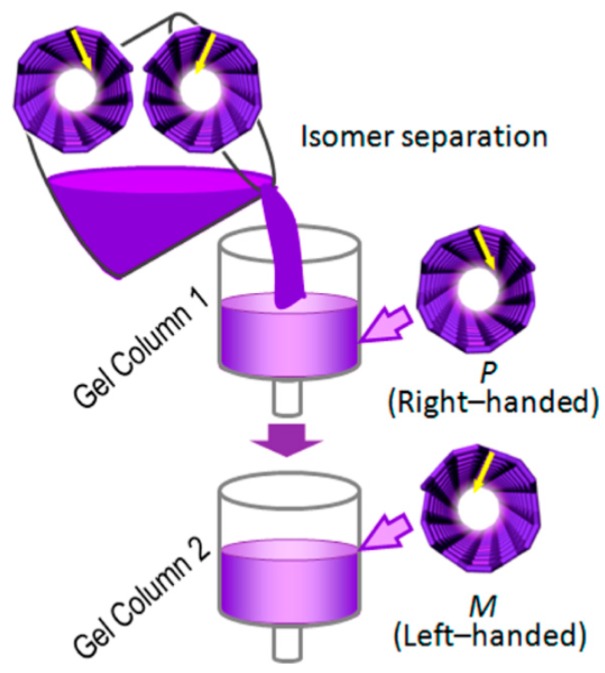
Illustration of the enantioseparation of *P*- and *M*-SWCNTs by gel permeation liquid chromatography on an allyl-dextran stationary phase. Reprinted with permission from [69]. Copyright (2014) American Chemical Society.

In single-walled carbon nanotubes (SWCNTs), a single layer graphene sheet is rolled-up into a tubular structure. The use of SWCNTs as selective stationary phases for the chromatographic separation of various classes of achiral compounds has been reviewed [1]. SWCNTs exist in three forms, i.e., two achiral structures (armchair and zig-zag) and a chiral structure. Chiral single-walled carbon nanotubes (*n*,*m*)-SWCNTs are characterized by *n,m-* indices, where *n* and *m* are coordination numbers of the carbon atoms in the hexagonal network. By convention, when *n* is greater than *m*, the species is right-handed or *P* (for positive), and when *m* is greater than *n*, the species is left-handed or *M* (for negative).

(*n*,*m*)-SWCNTs represent interesting supramolecular entities for the *reciprocal* chromatographic selector/selectand relationship. The enantiomers of nine pre-separated single chirality (*n*,*m*)-SWCNTs of varying enantiomeric purity were separated on Sephacryl gel containing allyl-dextran as the chiral selector by gel permeation chromatography (Figure 16) [69]. The successful enantioseparation was proved by their opposite CD-spectra. The enantiomeric purities are not known and they varied for different (*n*,*m*)-single chirality SWCNTs with the second eluted enantiomer being more enriched [69]. The fractionation of (*n*,*m*)-SWCNTs by countercurrent chromatography also led to chiral recognition [70]. The method was based on the partition of sodium deoxycholate (SDC) dispersed (*n*,*m*)-SWCNTs in a polyethylene glycol/dextran aqueous two-phase system. The *(n,m)*-dependent bimodal elution peak width of the chiral nanotubes were interpreted as enantiomeric recognition in the presence of chiral SDC and polymeric dextran [70]. Dextran represents a complex branched glucan (a polysaccharide made of d-glucose building blocks). By the *reciprocal* approach, nonracemic (*n*,*m*)-SWCNTs may be considered in the future as novel CSPs for the enantiomeric separation of lower linear dextrins (‘acyclodextrins’, maltooligosaccharides) which are readily available in both enantiomeric forms obtained from d- and l-glucose, respectively. Incidentally, acetylated/silylated dextrins with a different degree of oligomerization and devoid of a molecular cavity have been used as CSPs for the enantioseparation of derivatized amino acids and various underivatized racemic compounds by GC, thus highlighting the role of the polar external surface of the selector for chiral recognition in contrast to the well-established inclusion mechanism exerted by cyclodextrins [71].

Using molecular dynamics simulations, Raffaini and Ganazzoli investigated in a theoretical study the adsorption and denaturation of an oligopeptide formed by 16 chiral l-α-amino acids and having a helical structure in the native state on both the inner and the outer surface of the chiral (10,20)- and (20,10)-SWCNTs with an opposite handedness, and of the armchair (16,16)-SWCNT nanotube with a similar diameter for comparison [72]. The molecular simulations indicated that the enantioseparation of the chiral carbon nanotubes by chromatographic methods are feasible using oligopeptides of a sufficient length. It was also suggested that natural oligopeptides could not only be used to separate enantiomeric nanotubes, but in the *reciprocal* fashion also chiral nanotubes of single handedness could be considered as chiral selectors for racemic oligopeptides (Giuseppina Raffiani, personal communication, 2016).

Unspecified chiral (*n,m*)-SWCNTs were tested as CSPs for the enantioseparation of twelve classes of racemic pharmaceuticals by nano-LC [73,74]. The finding would require that the commercially available chiral (6,7)-SWCNT (carbon >90%, 0.7–0.9 nm diameter by fluorescence) obtained from Sigma-Aldrich was nonracemic or even enantiomerically pure. Neither the mode of the required enantiomeric enrichment of the synthetic racemic (6,7)-SWCNT nor its chiroptical data was reported. Some racemic compounds showed a deviation of the expected 1:1 peak ratio and no enantioseparation was noticed for the chiral (7,8)-SWCNTs. Also in another report of the continuous-flow enantioseparation of carvedilol with fluorescent detection on chiral carbon nanotubes, chiroptic data and enantiomeric compositions of the chiral selector were not mentioned [75]. As chiral SWCNTs contain equal amounts of left- and right-handed helical structures, their use as CSP *requires the separation of these non-superimposable mirror image forms* into single enantiomers of defined enantiomeric purity as outlined by Peng et al. [76].

In order to investigate whether the use of SWCNTs can improve enantioseparations on an existing CSP, comprised of the chiral nonracemic ionic liquid (*R*)-*N,N,N*-trimethyl-2-aminobutanol-*bis*(trifluoromethane-sulfon)imidate, two capillary columns, i.e., one containing the chiral ionic liquid alone and the other containing the chiral ionic liquid together with the SWCNTs were tested by GC [77]. It was shown that the capillary column containing SWCNTs improved the enantioselectivity of the chiral ionic liquid. The ‘nonchiral’ role of the SWCNT was due to an increase of the surface area of the inner capillary wall by formation of a layer with a skeletal network, leading the enhanced retention times, which improved the resolution factor *R_s_* for the enantiomers [77].

## 4. Chromatography of Fullerenes and the *Reciprocal* Principle

**Figure 17:**
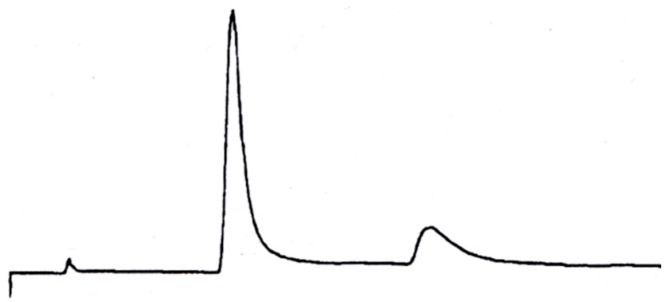
HPLC separation of C_60_ (12.2 min) and C_70_ (23.5 min) on a Pirkle-type ionically DNBPG containing column (250 × 10 mm I.D.) eluted with *n*-hexane at 5.0 mL/min and detected at 280 nm, α = 2.25 (r.t.). Reprinted with permission from [80]. Copyright (1990) American Chemical Society.

The molecular recognition of fullerene congeners on various selectors by liquid chromatography has been reviewed [1,78,79], including enantioseparation of chiral native fullerenes or its derivatives [1]. For more selective molecular recognition leading to large separation factors between C_60_ and C_70_ (α > 2), a supramolecular approach was first employed by Hawkins et al. [80]. As a strong π-acceptor selector for the π-donor fullerene selectands, a CSP containing *N*-(3,5-dinitrobenzoyl)-phenylglycine (DNBPG), originally developed by Pirkle et al. for enantioseparations [23], was employed (Figure 17).

**Figure 18:**
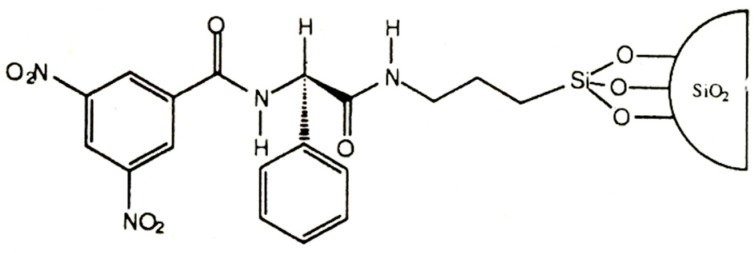
*N*-(3,5-dinitrobenzoyl)-phenylglycine (DNBPG) bonded to silica. Reprinted with permission from [81]. Copyright (1990) American Chemical Society.

An unprecedented increase in retention of C_60_ and C_70_ with increasing column temperature was observed by Pirkle and Welch on the covalently bonded π-acidic *N*-(3,5-dinitrobenzoyl)-phenylglycine (DNBPG) containing column (250 × 4.6 mm I.D.) (Figure 18) [81]. Van’t Hoff plots revealed the rare case of positive enthalpy and entropy values, i.e., the gain in entropy is accompanied by an endothermic adsorption.

**Figure 19:**
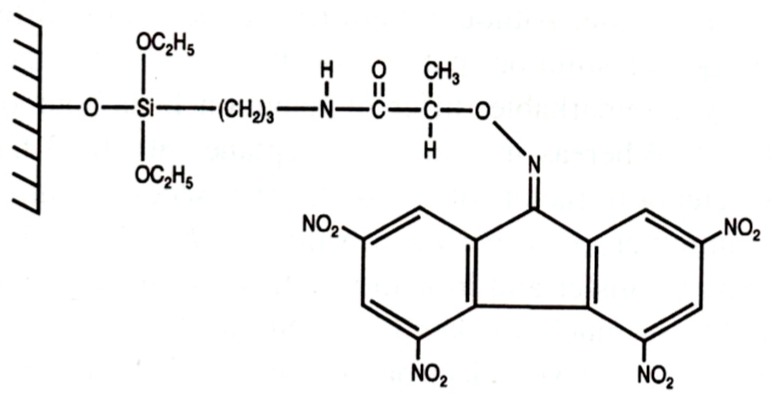
Structure of (*R*)-(−)-2-(2,4,5,7-tetranitro-9-fluorenylideneamino-oxy)propionic acid (TAPA) bonded to silica gel [4,84].

Tripodal 3,5-dinitrobenzoate ester and 2,4-dinitrophenyl ether stationary phases containing three π-acidic functionalities for simultaneous multipoint supramolecular interaction with fullerenes (‘Buckyclutcher’) were developed and they provided the highest retention and the greatest separation factor for the C_60_/C_70_ mixture [82]. A remarkable separation of 15 fullerene congeners in the range of C_60_–C_96_, including enantiomerically enriched specimens, by HPLC on a syndiotactic poly(methyl methacrylate) selector in 45 min with chlorobenzene as eluent has been described [83].

(*R*)-(−)-2-(2,4,5,7-tetranitro-9-fluorenylideneamino-oxy)propionic acid (TAPA) (Figure 1b and Figure 19), a strong π-electron acceptor due to the tetranitrofluorenylidene moiety, forms supramolecular charge transfer complexes with appropriate π-donor molecules. TAPA was used to separate C_60_ and C_70_ with a separation factor α up to 2.6 and the same peculiar increase of retention with increasing temperature previously observed with π-acidic *N*-(3,5-dinitrobenzoyl)-phenylglycine (DNBPG) was found and interpreted as arising from solute solvation at low temperatures [84]. The same authors mentioned that C_60_ and C_70_ can behave as both π-electron donor and π-electron acceptor (the electron affinity of C_60_ amounts to 2.6 eV).

**Figure 20:**
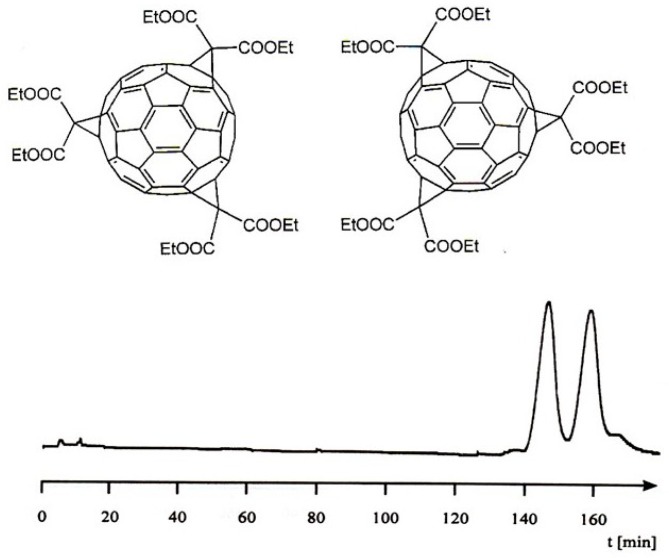
**Top**: Structure of the *tris*-adduct C_60_[C(COOEt)_2_]_3_. **Bottom**: Enantioseparation of a *hexakis*-adduct C_60_[C(COOEt)_2_]_6_ on the CSP TAPA bonded to aminopropyl silica gel by micro HPLC (α = 1.1). Packed fused-silica capillary (20 cm × 0.25 mm I.D.), μL/min acetonitrile/water (75/25, *v/v*), room temperature. Reprinted with permission from [85].

The enantioseparation of the *tris*-adduct C_60_[C(COOEt)_2_]_3_ and *hexakis*-adduct C_60_[C(COOEt)_2_]_6_ entailed with an inherent chiral substitution pattern (Figure 20) has also been described on the CSP (*R*)-(−)-2-(2,4,5,7-tetranitro-9-fluorenylideneaminooxy)propionic acid (TAPA) bonded to silica gel (Figure 19) by micro HPLC [85].

The enantioseparation of *bis*-, *tris*- and *hexakis*-adducts of C_60_ (Figure 20, top) has been achieved by HPLC on the Whelk-O1-CSP [86]. As the Whelk-O1-CSP (Figure 3c) was originally optimized for racemic naproxen [23], it may be speculated that some *bis*-, *tris*- and *hexakis*-adducts of C_60_ may also be enantioseparated by the silica-bonded naproxen selector (Figure 3a) via a *twofold reciprocal* recognition scenario.

**Figure 21:**
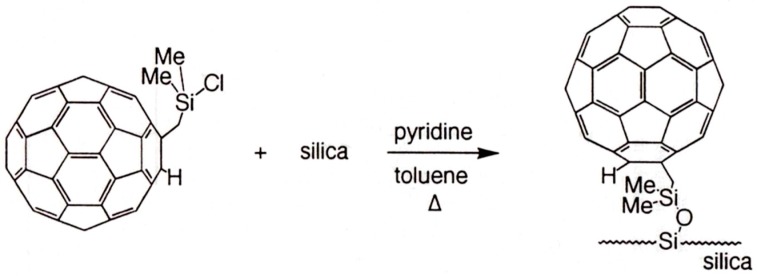
Synthetic scheme for a C_60_ bonded silica stationary phase. Reproduced with permission from [87]. Copyright Wiley-VCH, Weinheim, Germany.

In order to optimize supramolecular recognition between fullerenes and polyaromatic compounds (PAHs), the *reciprocal* principle of changing the chromatographic roles of selector and selectand has been suggested by Saito et al. [87]. In liquid chromatography, fullerene selectors can be used as supramolecular stationary phases either as native crushed specimen packed into capillaries or covalently linked to a functionalized silica gel [78,88,89]. The chemically bonded C_60_ phase (Figure 21) [87] showed preferential interaction with triphenylene and perylene which possess partial structures similar to that of C_60_. Interestingly, larger retention factors are observed for nonplanar vs. planar PAH molecules of comparable molecular size. Thus effective molecular recognition occurs between the C_60_ selector and nonplanar selectands with a similar molecular curvature of the fullerene [87]. Bonded C_60_ phases were also used for self-recognition, i.e., for the congener separation of C_60_ and C_70_ [87,88]. As compared to an octadecylsilica (ODS) reversed phase devoid of aromatic moieties, a C_60_ phase entailed high retention factors for C_60_ and C_70_ and an enhanced separation factor (α = 2.9) [87].

Fullerenes have also been used as stationary phases in gas chromatography (GC). A glass capillary column (15 m × 0.29 mm I.D.) was coated with pure C60 (film thickness 0.1 μm) using a high-pressure static method [90]. The thermostable solid C60 phase behaved like the liquid phase squalane in its van der Waals interaction with *n*-alkanes.

**Figure 22:**
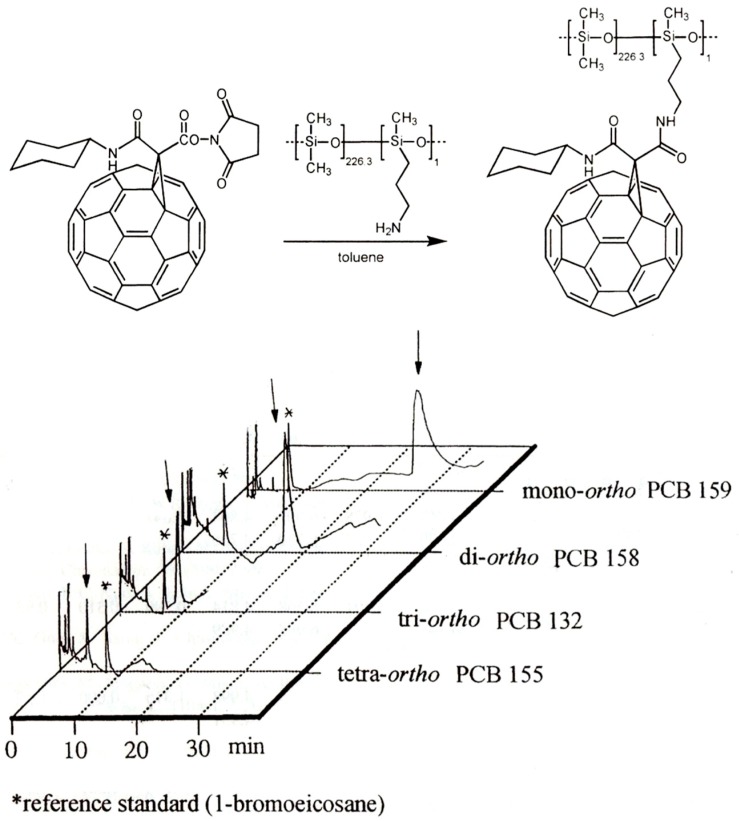
**Top**: Synthesis of C_60_ linked to aminopropyl poly(dimethylsiloxane). **Bottom**: Gas chromatographic elution order of hexachlorobiphenyl congeners with different degree of *ortho*-chloro substitution. The increased retention time of the second eluted enantiomer is marked with an arrow. 10 m × 0.25 mm I.D. fused silica column coated with the C_60_ phase (0.25 μm), 190 °C, carrier: 0.6 bar (at gauge) helium, electron capture detection. Reprinted with permission from [93].

C_60_ linked to poly(dimethylsiloxane) (Figure 22, top) was coated on a 10 m × 0.25 mm I.D. fused silica column and used for the selective separation of isomeric hexachlorobiphenyls differing in *ortho*-chlorine substitution pattern. Thermodynamic parameters were obtained by the retention-increment method *R*′ [91,92]. It separates supramolecular interactions from non-specific interactions of the linker. It was shown that the retention behaviour of PCBs depends strongly on the degree of *ortho*-chlorine substitution which in turn is linked with non-planarity (Figure 22, bottom). The deviation from planarity of PCBs strongly decreases the supramolecular interaction with C_60_ [93]. Since planar PCBs are more toxic than non-planar PCBs, a tentative link of retention behaviour on C_60_ and toxicity has been advanced [93].

In Table 4 the selective interaction of aromatic compounds with C_60_ linked to poly(dimethylsiloxane) (Figure 22, top) is expressed by the retention-increment *R′*, i.e., a value of two indicates that the retention of the PCB congener on C_60_ is twice as compared to a reference column devoid of the fullerene selector [94].

## 5. The *Reciprocal* Selectand/Selector System of Fullerenes/Cyclodextrins

**Figure 23:**
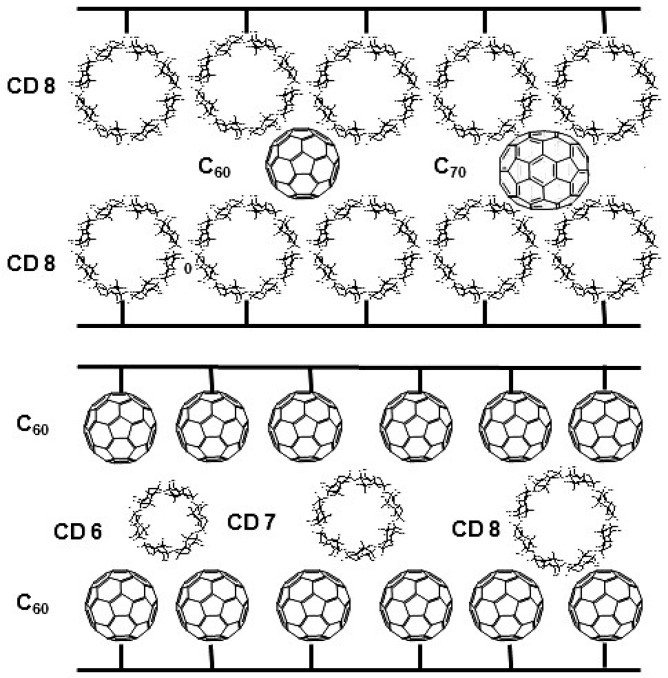
Schematic representation of the *reciprocal* principle between fullerenes and cyclodextrins. Reproduced from A. Bogdanski, Doctoral Thesis, University of Tübingen, Tübingen, Germany, 2007.

A supramolecular selectand/selector system *par excellence* is represented by fullerenes (C*_n_*, *n* = number of carbon atoms) and cyclodextrins (CD*n*, *n* = number of d-glucose building blocks). It also lends itself to the principle of *reciprocal* recognition. According to Figure 23, the role of selector and selectand can be reversed.

The non-chromatographic supramolecular recognition phenomenon between fullerenes and cyclodextrins is well established. γ-Cyclodextrin (CD8) forms water-soluble bicapped 2:1 association complexes with [60]fullerene (C_60_) [95,96,97,98,99,100,101]. Also β-cyclodextrin (CD7) may undergo complexation with C_60_ [102,103].

**Figure 24:**
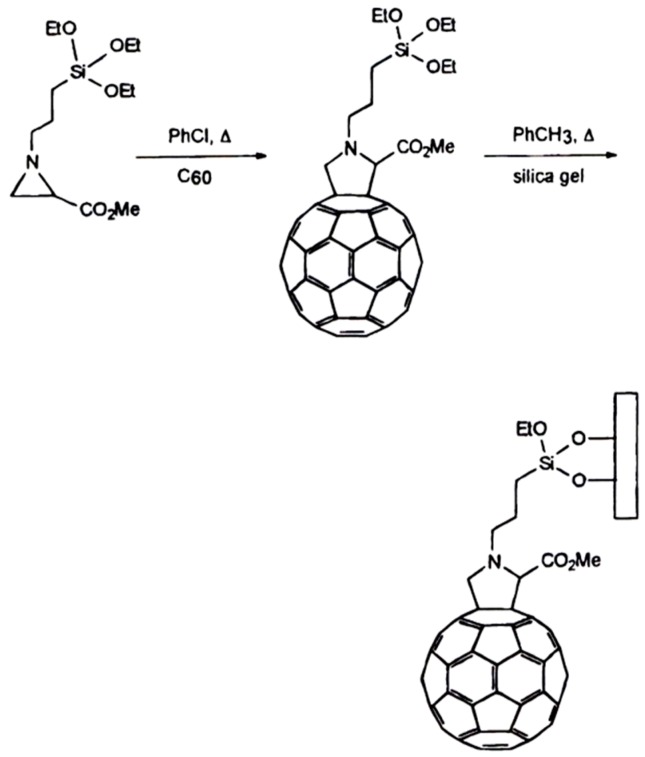
Synthetic pathway to a 6,6-[60]fulleropyrrolidine derivative by aziridine ring opening via 1,3-dipolar addition to C_60_ and grafting the fullerene-containing triethoxysilane to silica microparticles leading to a chemically homogeneous material with defined structure and high chromatographic efficiency. Reprinted with permission from [105]. Copyright (1997) American Chemical Society.

**Figure 25:**
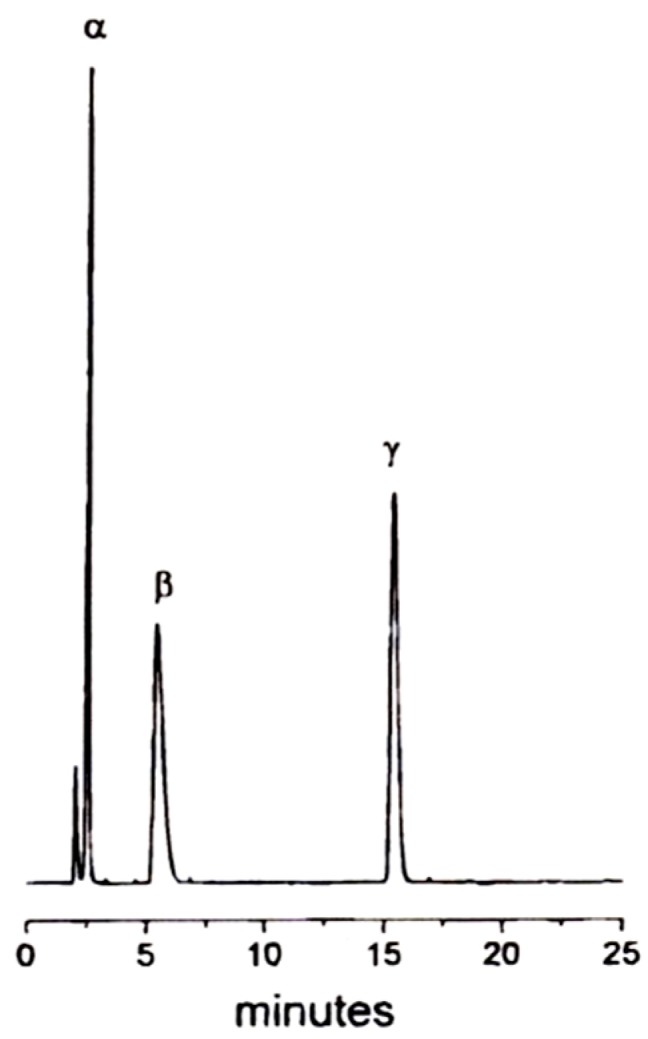
Separation of α-, β- and γ-cyclodextrins (CD6, CD7, CD8) on immobilized C_60_ by HPLC. 250 × 1.8 mm I.D. stainless steel column packed with [60]fulleropyrrolidine-based silica (Hypersil, 5 μm), eluent: 0.25 mL/min water/methanol/tetrahydrofuran (80/10/10 for 3 min and then linear gradient to 40/30/30 in 25 min, at 25 °C. Reprinted with permission from [105]. Copyright (1997) American Chemical Society.

Shape selectivity governs the HPLC separation of C_60_ and C_70_ on native γ-cyclodextrin chemically bonded to silica [104]. C_70_ was much more strongly retained than C_60_ on this stationary phase whereas no separation of the two fullerenes occurred on the corresponding unmodified silica indicating that the separation is due to the selective supramolecular interaction with the cyclodextrin selector [104]. In a *reciprocal* strategy, Bianco et al. used a C_60_-bonded phase (Figure 24) in an aqueous/organic medium for the high-efficiency HPLC separation of α-, β- and γ-cyclodextrins (CD6-CD8) [105]. As expected, the trace shows increased retention with increased molecular weight of CD*n* (Figure 25).

**Figure 26:**
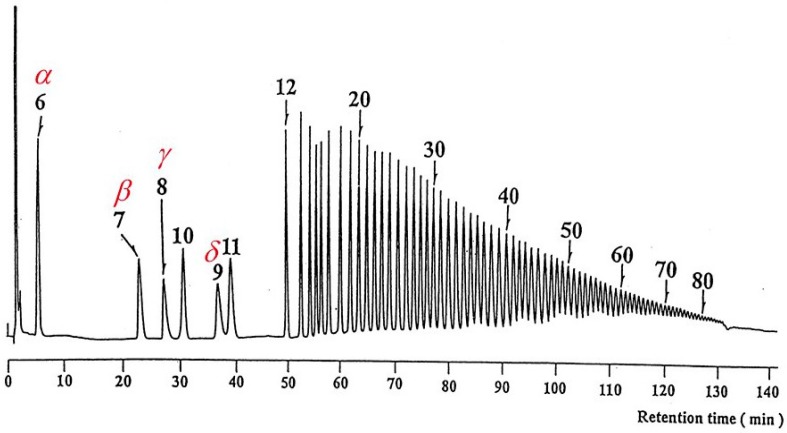
Separation of cyclodextrin congeners (CD6–CD85) enzymatically obtained by cyclodextrin glycosyltransferase from *Bacillus macerans* on synthetic amylose by high-performance anion-exchange chromatography (HPAEC) with pulsed amperometric detection using a 25 cm × 4 mm I.D. Dionex CarboPac PA-100 column. Reprinted with permission from [108].

With the advent of the enzymatic access to large-ring cyclodextrins [106,107], separation of the congeners CD*n* represented a considerable analytical challenge. Koizumi et al. separated CD6-CD85, obtained by the action of cyclodextrin glycosyltransferase from *Bacillus macerans*, on synthetic amylose by high-performance anion-exchange chromatography (HPAEC) with pulsed amperometric detection using a 25 cm × 4 mm I.D. Dionex CarboPac PA-100 column (Figure 26) [108], whereas Bogdanski et al. separated CD6-CD21 by liquid-chromatography with electrospray ionization mass spectrometric detection (LC/ESI-MS) using a 25 cm × 4 mm I.D. LiChrospher NH_2_ column [109]. With one exception (CD9 on HPAEC) the congeners CD*n* were eluted from the stationary phases according to the degree of polymerization (Figure 26).

**Figure 27:**
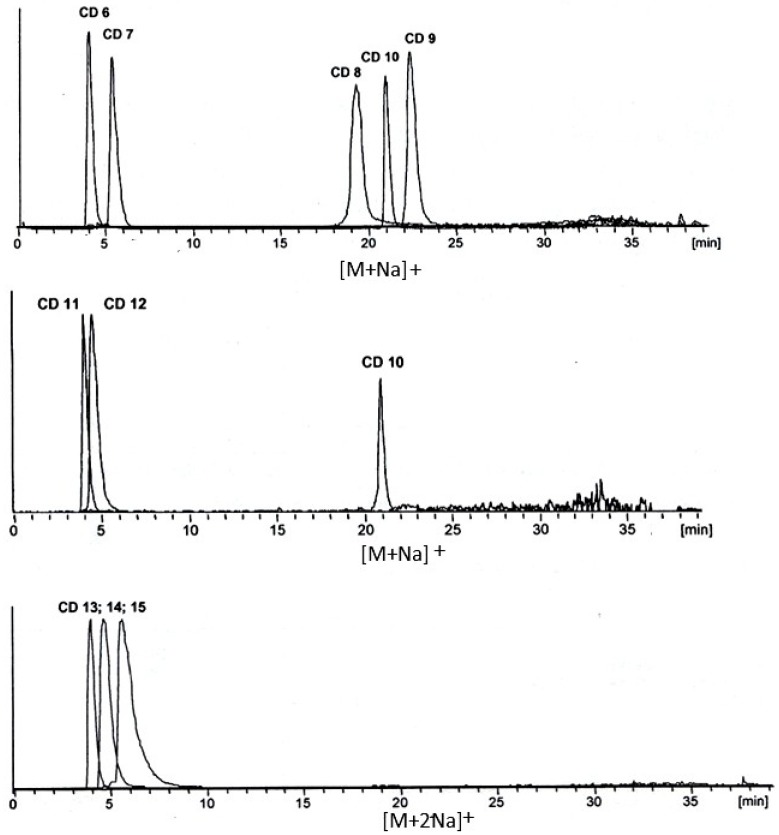
Overlaid LC-ESI-MS traces of the selective separation of CD6-CD15 sodium ion adducts on C_60_ on a 250 × 4 mm I.D. stainless steel column packed with silica-bonded C_60_ stationary phase, 0.5 mL/min water to acetonitrile/tetrahydrofuran gradient elution, 25 °C [110]. Reproduced from A. Bogdanski, Doctoral Thesis, University of Tübingen, Tübingen, Germany, 2007.

In striking contrast, an unusual supramolecular selectivity pattern has been observed for the *reciprocal* separation of CD6–CD15 congeners on a fullerene C_60_ selector anchored to silica particles (Figure 27), which strongly deviates from the usually observed correlation of retention and molecular weight [110]. Thus, whereas CD6 and CD7 exhibit a low retention and are eluted in ~5 min, CD8–CD10 are eluted only after a long retention gap of about 15 min with the elution order CD8 < CD10 < CD9 in ~20 min. The higher CD11-CD15 congeners are eluted together with CD6 in ~5 min (Figure 27). The drop of retention between CD10 and CD11 of 15 min is indeed remarkable. The unusual elution order has been detected with LC-electrospray ionization-mass spectrometry (ESI-MS) of sodium ion adducts of CD6-CD15. The results were confirmed for CD6-CD25 by employing an LC-evaporative light scattering detection (ELSD) system [110].

**Figure 28:**
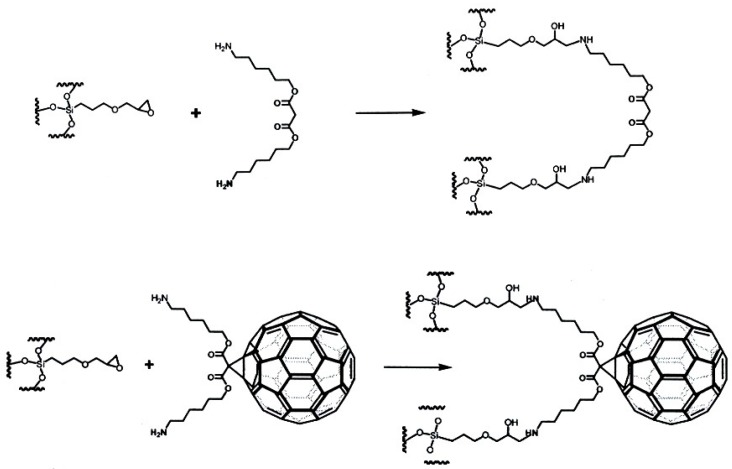
Structure of the silica-bonded spacer stationary phase (**top**) and the silica-bonded C_60_ stationary phase (**bottom**) [110]. Reproduced from A. Bogdanski, Doctoral Thesis, University of Tübingen, Tübingen, Germany, 2007.

The retention-increment method [91,92] has been employed to quantify the unusual selectivity. It separates supramolecular interactions from non-specific interactions of the linker. Thus two stationary phases, i.e., one containing the spacer alone and one containing the C_60_ selector bonded via the spacer were prepared (Figure 28). Nucleosil (5 μm, 100 Å) was reacted in toluene with 3-glycidoxypropyltrimethoxysilane to yield 3-glycidoxypropyl-silica and (i) the epoxide was reacted with *bis*(1-aminohexyl)malonate to the silica-bonded spacer without selector and (ii) the same epoxide was reacted with *bis*(1-aminohexyloxycarbonyl)malonate-dihydro[60]fullerene [111]. The striking selectivity change of CD*n* occurred only on the C_60_-selector but not on the silica-bonded spacer devoid of the C_60_ selector. Due to the necessary use of an LC elution gradient only *apparent* relative complexation constants K_rel_ (related to CD6) were obtained (Table 5). They nevertheless highlight the remarkable stability differences for the CD6-CD12 congeners in their supramolecular interaction with C_60_ [110]. The striking selectivity pattern (Table 5) may be subject to molecular modelling studies in the future. The well-known deviation from the ideal ‘doughnut’ structure of small CD*n* (*n* < 10) due to conformationally strain-induced band flips and kinks in CD*n* (*n* > 10) [112] may reveal clues of molecular recognition pattern.

It has been speculated [110] that by using a single [78]fullerene selector (C_78_), the most strongly interacting CD-congener can be identified and, according to the *reciprocal* principle, could then applied as a silica-bonded resolving agent for the separation of all five isomeric [78]fullerenes including the direct enantioseparation of chiral *D*_3_-[78]-fullerene [113].

## 6. Chromatography of Calixarenes and the *Reciprocal* Principle

**Figure 29:**
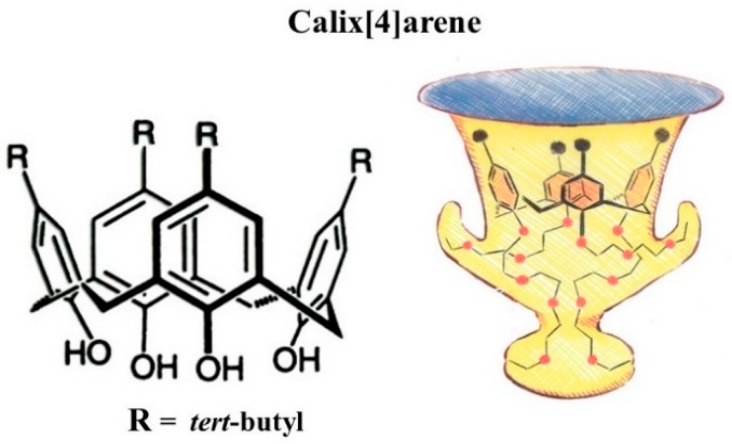
The structure of calix[4]arene. Reproduced with permission of the Royal Society of Chemistry from [114].

Gutsche reviewed the application of calixarenes (Figure 29) as *reciprocal* selectors and selectands [114].

**Figure 30:**
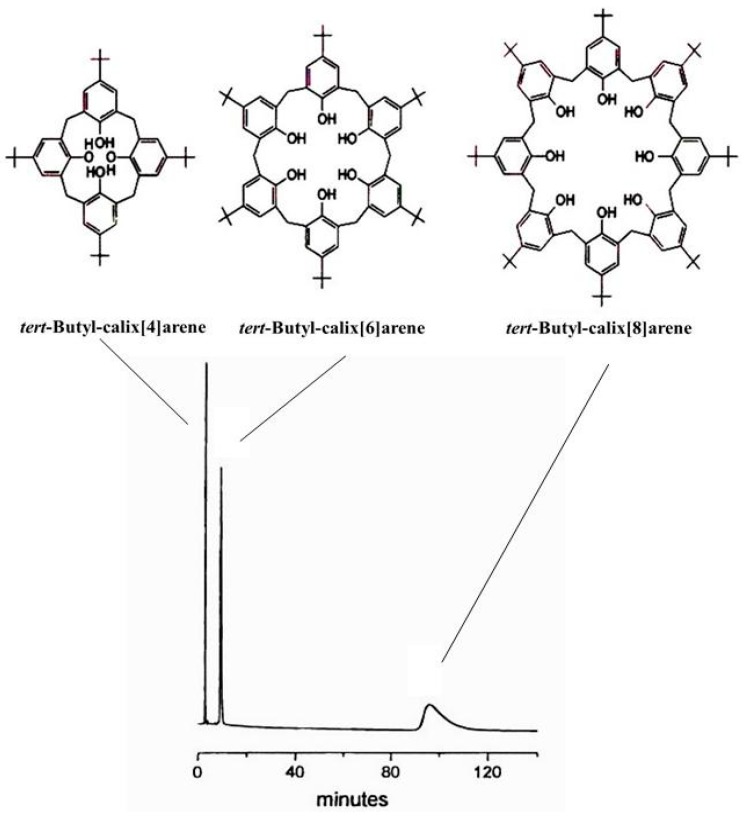
**Top**: Structures of *tert*-butyl-calix[*n*]arenes. **Bottom**: HPLC trace for the separation of *tert*-butylcalix[*n*]arenes on a 6,6-[60]fulleropyrrolidine stationary phase (Figure 24). 250 × 1.8 mm I.D. stainless steel column. Eluent: CH_2_Cl_2_/2-propanol (99.5/0.5), flow rate 0.3 mL/min; T: 25 °C; UV detection at 280 nm. Reprinted with permission from [105]. Copyright (1997) American Chemical Society.

Following the purification of C_60_- and C_70_-fullerenes with calix[8]arene [115,116], a *reciprocal* separation of calix[4]arene, calix[6]arene and calix[8]arene (Figure 30, top) by microcolumn liquid chromatography (μ-LC) on a chemically-bonded C_60_ silica stationary phase (Figure 21) has been reported by Saito et al. [117]. An improved sseparation of the same calixarene congeners by μ-LC on a 6,6-[60]fulleropyrrolidine silica stationary phase (Figure 24) was later reported by Bianco et al. (Figure 30, bottom) [105].

Achiral chromatographic selectivity has been achieved for various amino acid esters on silica-bonded calix[4]arene tetraester [118,119]. The enantioseparation of binaphthyl atropisomers on the chiral acylcalix[4]arene amino acid derivatives (*N*-l-alaninoacyl)calix[4]arene and (*N*-l-valinoacyl)calix[4]arene) by electrokinetic chromatography (EKC) has been reported [120]. By enantioselective liquid chromatography a chiral calixarene functionalized with (−)-ephedrine at the lower rim and chemically bonded to silica has been used to separate racemic 1-phenyl-2,2,2-trifluoroethanol [121].

**Figure 31:**
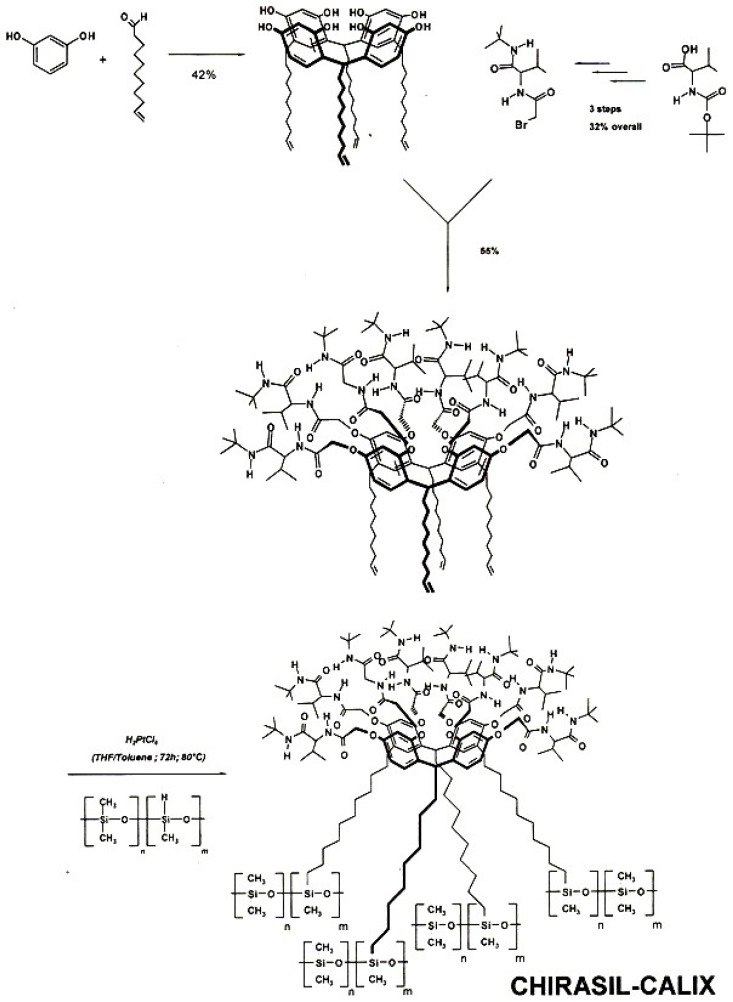
Synthesis of the CSP Chirasil-Calix. Reprinted with permission from [123].

A chiral resorc[4]arene selector [122] of the basket-type containing four ω-unsaturated alkyl chains was synthesized from resorcinol and 1-undecenal [123]. Afterwards chiral l-valine-*tert*-butylamide moieties were attached to the eight hydroxyl groups. The chiral resorc[4]arene was then linked via the ω-unsaturated alkyl chains to poly(dimethyl-methylhydro)siloxane via platinum-catalyzed hydrosilylation to yield chemically bonded Chirasil-Calix-Val (Figure 31) [123]. On a 20 m × 0.25 mm I.D. fused silica capillary column coated with Chirasil-Calix (0.25 μm) the *N*(*O*,*S*)-trifluoroacetyl-d,l-α-amino acids Ala, Abu, Val, Ile, Leu, Pro, Phe, Glu, Tyr, Orn and Lys were enantioseparated by GC with *R_s_* > 2 [122].

**Figure 32:**
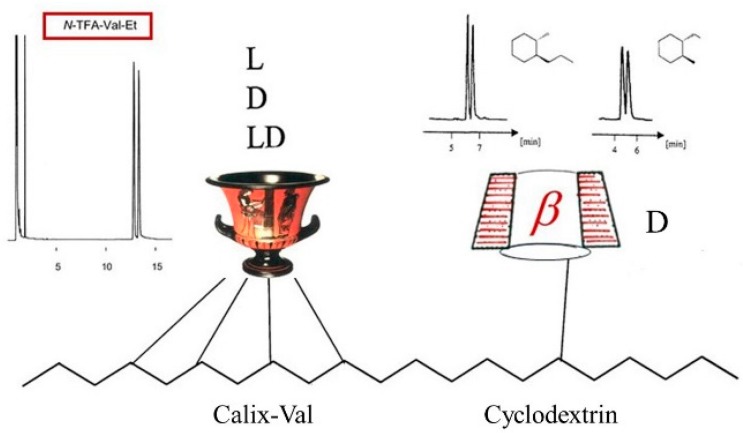
The mixed CSP Chirasil-Calix-Val-Dex.

In the *dual* chiral recognition system *Chirasil-Calix-Val-Dex*, a resorc[4]arene containing l-valine diamide (Calix-Val) *and* permethylated β-cyclodextrin (CD) were chemically linked to poly(dimethylsiloxane) [124]. The selector system belongs to the category of *mixed* chiral stationary phases [125]. On this binary CSP both *N*-TFA-valine ethyl ester (due the presence of Calix-Val) and chiral unfunctionalized 1,2-*trans*-dialkylcyclohexanes (due to the presence of the CD) were simultaneously enantioseparated (Figure 32) [124]. In order to account for *matched* and *mismatched* enantioselectivities imparted by the two selectors, the chirality of Calix-Val could be changed from to l to d. When racemic Calix-LD-Val were used, any observed enantioselectivity could only arise from the presence of CD [124].

**Figure 33:**
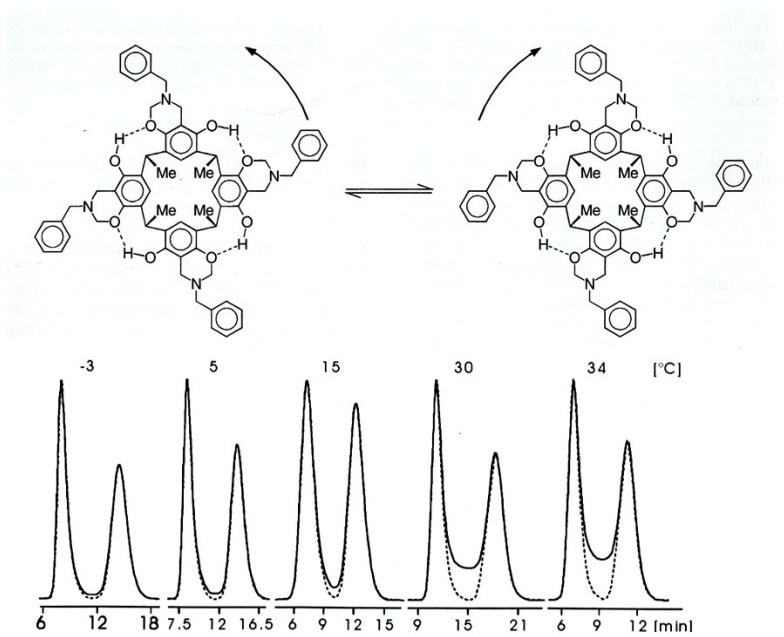
**Top**: Interconverting enantiomers of tetrabenzoxacine resorc[4]arene by HPLC on the CSP Chiralpak AD. **Bottom**: Distorted peak profiles caused by enantiomerization (dotted line: expected peak profiles devoid of interconversion). Reprinted with permission from [127].

In the example above, the chiral resorc[4]arene is used as an enantioselective selector. In a reversed fashion the enantiomers of a chiral resorc[4]arene compound can also be studied as selectands. Thus, tetrabenzoxacine resorc[4]arene (Figure 33, top) was enantioseparated by HPLC on the CSP Chiralpak AD developed by Okamoto et al. [126]. The chromatograms show temperature-dependent interconversion profiles due to *enantiomerization* during enantioseparation (Figure 33, bottom) [127]. By peak-form analysis featuring plateau formation [128], the inversion barrier was determined to Δ*G* = 92 ± 2 kJ/mol (298K) [127].

This finding is recalled here, because one may speculate to employ interconverting calixarene enantiomers in a *reciprocal* fashion as a dynamic CSP in the spirit of a scenario of column deracemization described by Welch [129]. Thus when an enantiomerically pure selectand, which interacts with high affinity and enantioselectivity with an interconverting selector present in the stationary phase, is added to the mobile phase, it may cause deracemization of the selector, thereby creating a nonracemic CSP for subsequent enantiomeric separations of the selectand in its racemic form. This intriguing scenario was demonstrated by HPLC with a conformationally labile naphthamide atropisomer [129]. First it was shown that the naphthamide can be enantioseparated on the Whelk-O1 CSP (Figure 3c). Under stopped-flow conditions [130], deracemization took place in the mobile phase. With the *reciprocal* concept the racemic naphthamide was then linked to silica whereas the soluble analog of the enantiomerically pure Whelk-O1 selector was added to the mobile phase. After deracemization of the CSP, the racemic analog of the Whelk-O1 selector was enantioseparated, however, with the expected subsequent decrease of the separation factor α due to gradual racemization of the CSP [129].

The *reciprocal* concept of interconverting molecules in separation methods could also be extended to the liquid chromatographic investigation of conformational *cis/trans*-diastereomers of l-peptidyl-l-proline. Studies of proline-containing peptides separated on calixarene-bonded silica gels have been investigated by Gebauer et al. [131] and interconversion barriers of l-proline containing dipeptides by dynamic CE were determined [132].

## 7. Conclusions

Since the first reports by Mikeš and Pirkle on the use of a *reciprocal* approach in chromatography through changing the role of selectands and selectors, many examples have been reported in different areas of selective supramolecular interactions including combinatorial advances. Advantages and limitations of the *reciprocal* concept are outlined. The *reciprocal* strategy clearly aids the development of optimized selectors for difficult separations of selectands including enantiomers. Various scenarios of the interchanging role of selectors and selectands in separation science are discussed in the hope that this will stir further endeavors devoted to the principle of *reciprocity* in supramolecular recognition.

The present account should be seen as part of the emerging field of supramolecular separation methods [1,2] with further emphasis also to molecular sensor approaches and surface related recognition phenomena. The optimization of selector/selectand systems is the precondition for meaningful mechanistic studies by various molecular modelling approaches.

## Figures and Tables

**Table 1:** List of sublibraries of cyclohexapeptides used in the deconvolution process. X stands for one of the 18 of the common l-α-amino acids except cysteine and tryptophan. Reprinted with permission from [45]. Copyright (1998) American Chemical Society.

Numbering	Cyclohexapeptide Library
S.1.1:	*cyclo*(Arg-Lys-X-X-X-β-Ala)
S.2.1:	*cyclo*(Arg-Lys-Pro-X-X-β-Ala)
S.2.2:	*cyclo*(Arg-Lys-X-Pro-X-β-Ala)
S.2.3:	*cyclo*(Arg-Lys-X-X-Pro-β-Ala)
S.3.1:	*cyclo*(Arg-Lys-Val-Pro-X-β-Ala)
S.3.2:	*cyclo*(Arg-Lys-Met-Pro-X-β-Ala)
S.3.3:	*cyclo*(Arg-Lys-Ile-Pro-X-β-Ala)
S.3.4:	*cyclo*(Arg-Lys-Leu-Pro-X-β-Ala)
S.3.5:	*cyclo*(Arg-Lys-Tyr-Pro-X-β-Ala)
S.3.6:	*cyclo*(Arg-Lys-Phe-Pro-X-β-Ala)
S.4.1:	*cyclo*(Arg-Lys-Tyr-Pro-Val-β-Ala)
S.4.2:	*cyclo*(Arg-Lys-Tyr-Pro-Met-β-Ala)
S.4.3:	*cyclo*(Arg-Lys-Tyr-Pro-Ile-β-Ala)
S.4.4:	*cyclo*(Arg-Lys-Tyr-Pro-Leu-β-Ala)
S.4.5:	*cyclo*(Arg-Lys-Tyr-Pro-Tyr-β-Ala)
S.4.6:	*cyclo*(Arg-Lys-Tyr-Pro-Phe-β-Ala)

**Table 2:** Resolution factors *R_s_* of DNP-amino acids on cyclohexapeptides by CE (conditions as in Figure 6) [49].

Amino Acid/Cyclopeptide	DNP-Glu	DNP-Norleu	DNP-Meth	DNP-Ala	DNP-Norval	DNP-Threo
*cyclo*(Arg-Lys-Tyr-Pro-Tyr-β-Ala)	4.97	3.80	5.46	2.27	3.64	2.10
*cyclo*(Arg-Lys-Phe-Pro-Phe-β-Ala)	2.33	1.63	1.96	1.03	1.66	0.59
*cyclo*(Arg-Lys-Trp-Pro-Trp-β-Ala)	1.69	1.31	1.21	1.47	1.93	0.90

**Table 3:** Resolution factors *R_s_* of DNP-amino acids on cyclopeptides of varying ring size by CE (conditions as in Figure 6) [49].

Amino Acid/Cyclopeptide	DNP-Glu	DNP-Norleu	DNP-Meth	DNP-Ala	DNP-Norval	DNP-Threo
*cyclo*(Arg-Lys-Tyr-Pro-Tyr-β-Ala)	4.97	3.80	5.46	2.27	3.64	2.10
*cyclo*(Lys-Lys-Tyr-Tyr-Tyr-Tyr-Lys)	0.36	1.41	1.53	1.05	1.46	0
*cyclo*(Lys-Tyr-Arg-Tyr-β-Ala)	0	0.62	0	0.35	0.37	0
*cyclo*(Arg-Lys-Tyr-Tyr-β-Ala)	0.23	0.73	0.43	0.34	0.55	0

**Table 4:** Retention-increments *R′* for the selective interaction of aromatic compounds with C_60_. Complexation column (*k*): 10 m x 0.25 mm I.D. fused silica column coated with the C_60_ phase (0.25 μm). Reference column (*k*_0_, without C_60_): 10 m × 0.25 mm I.D. fused silica column coated with dimethyl-acetylaminopropyl(methyl)polysiloxane (0.25 μm) [94].

Analyte	T (°C)	*R′* = (*k* − *k*_0_)/*k*_0_
1.2-Dichlorobenzene	80	0.61
2.6-Dichlorotoluene	80	0.69
Nitrobenzene	80	0.95
2-Nitrotoluene	80	0.91
Aniline	80	1.58
Phenol	90	0.50
Naphthalene	90	0.73
1-Methylnaphthalene	120	0.83
2-Methylnaphthalene	120	0.70
Indole	120	1.28
Quinoline	120	1.87
Isoquinoline	120	2.94
4-Chlorophenol	120	0.34
2.4-Dichlorophenol	120	0.77
2.4.6-Trichlorophenol	120	1.29

**Table 5:** Apparent complexation constants K_rel_ of cyclodextrins and silica-bonded [60]fullerene (C_60_) related to CD6 (α-cyclodextrin) [110].

	CD6	CD7	CD8	CD9	CD10	CD11	CD12
K_rel_	1	4.5	39	46	42	1	2
